# Large‐Scale Genomics Reveals Three‐Source Ancestry and Layered Adaptation to High Altitude in Tibetan Chickens

**DOI:** 10.1002/advs.202522994

**Published:** 2026-05-12

**Authors:** Zongyi Zhao, Ruixue Nie, Hailu Fan, Tenzin Ngodroup, Li Zhu, Hongbin Pan, Bo Zhang, Hao Zhang

**Affiliations:** ^1^ Frontiers Science Center for Molecular Design Breeding (MOE) China Agricultural University Beijing China; ^2^ State Key Laboratory of Animal Biotech Breeding College of Animal Science and Technology China Agricultural University Beijing China; ^3^ Inner Mongolia Herbivorous Livestock Feed Engineering Technology Research Center Hohhot China; ^4^ Animal Disease Prevention and Control Center of Xizang Autonomous Region (Animal Husbandry Station of Xizang Autonomous Region) Lhasa China; ^5^ Faculty of Animal Science and Technology Yunnan Agricultural University Kunming China

**Keywords:** high‐altitude adaptation, human‐mediated gene flow, population genomics, Tibetan chicken

## Abstract

Tibetan chickens exhibit adaptive traits for the hypoxic Tibetan Plateau, yet how distinct ancestral inputs, their timing, and their functional consequences jointly shape this adaptation remains poorly understood. To address this, we integrated admixture modeling with ancestry tract‐length dating across 1054 whole genomes, resolving three distinct ancestral sources—Northwest China (NWC), the Sichuan–Yunnan adjacent region (SYA), and the Southern Himalayan Foothills (SHF)—whose contributions are temporally stratified: NWC forms the deepest founding layer (>928 generations), SHF records an ancient but low‐intensity signal (∼928 generations; 95% CI: 875–1024), and SYA reflects a major recent expansion (∼514 generations; 95% CI: 493–541) with stepwise diffusion across the plateau. Selection scans calibrated against a demographic null model indicated that these sources are enriched for distinct functional categories: NWC for vascular homeostasis and coagulation (e.g., *VWF, TSPAN9*), SYA for calcium signaling and metabolic regulation (e.g., *CACNA2D1, AMY2A*), and SHF for pulmonary vascular remodeling (e.g., *AGTR1*). These findings indicate that high‐altitude adaptation in Tibetan chickens involves temporally layered contributions from multiple ancestral sources, each associated with distinct candidate functional pathways—a pattern consistent with human‐mediated dispersal shaping the genetic architecture of highland populations.

## Graphical abstract

Whole‐genome analysis of 1,054 chickens reveals three ancestral sources (NWC, SYA, and SHF) with distinct temporal entry patterns into the Tibetan Plateau. Route‐specific selection scans, calibrated against a demographic null, suggest complementary functional enrichments—vascular homeostasis (NWC), calcium signaling and cardiac adaptation (SYA), and pulmonary vascular remodeling (SHF)—pointing to a temporally layered, human‐mediated assembly of adaptive genetic components.

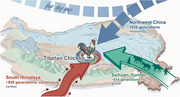

## 1 Introduction

The complex interplay between natural and artificial selection has profoundly shaped the evolutionary trajectory of domesticated animals. Charles Darwin once described the process of animal and plant domestication as “one grand experiment in evolution” [1]. Among the most extreme settings for this experiment is the Qinghai–Tibet Plateau, where chronic hypoxia, intense ultraviolet radiation, and large diurnal temperature swings impose relentless selective pressure [2]. Over millennia, human migration, trade, and shifting preferences have intersected with these harsh environmental forces, creating conditions under which domesticated animals have faced sustained physiological challenges for thousands of years [3, 4]—an ongoing process whose signatures are now detectable in their genomes. Comparative studies in plateau mammals—including yaks [5, 6], Tibetan antelopes [7, 8, 9], and Tibetan pigs [10, 11]—have documented convergent or lineage‐specific solutions to extreme environments. These findings have informed mechanistic hypotheses about the genetic basis of high‐altitude adaptation [12, 13, 14]. However, these insights derive primarily from wild or semi‐wild taxa whose genomes have been sculpted chiefly by natural selection over deep evolutionary timescales. For domesticated species, the picture may be fundamentally different: their genomes bear the simultaneous imprint of human‐mediated gene flow and artificial selection, raising the question of whether—and how—domestication reshapes the genetic architecture of high‐altitude adaptation [1, 15, 16, 17].

This distinction is pivotal, as domestication introduces evolutionary forces that are largely absent in wild taxa. Wild species adapted to high altitude typically through allele‐frequency shifts under sustained environmental pressure [7, 18, 19]. Domesticated livestock populations, by contrast, are repeatedly reshaped by human‐directed movements—trade, transhumance, and breed improvement—that inject genetic material from multiple, sometimes distant source populations [3, 19, 20, 21]. As a result, domesticated breeds must not only cope with plateau stressors but also integrate potentially heterogeneous ancestral inputs that arrive at different times and via different geographic corridors. In livestock and poultry, the interplay of ecological filtering and anthropogenic pressures creates signatures across modules relevant to performance and resilience, including metabolism, cardiovascular development, immune regulation, and stress responses [22, 23, 24]. These dual pressures suggest that high‐altitude adaptation in domestic species may be better understood as a multi‐source assembly rather than a single‐lineage accumulation—but this hypothesis remains largely untested.

The Tibetan chicken (*Gallus gallus domesticus*), which has been maintained on the Tibetan Plateau for centuries, offers an informative model for disentangling natural and human‐mediated selection. Historically, performance observations under hypoxia, thermoregulatory challenges, and reproductive output have suggested physiological adaptations to high altitudes [25, 26, 27, 28]. Recent genomic studies have begun to map this biology, highlighting polygenic architectures spanning vascular and calcium‐signaling genes, metabolic regulators, hemoglobin clusters, and hypoxia‐responsive transcription factors, converging on pathways plausibly tied to oxygen transport, cardiac function, and energetic homeostasis [26, 28, 29, 30, 31, 32, 33, 34, 35, 36, 37, 38].

Yet two key gaps limit current understanding. First, several studies have implicitly treated Tibetan chickens as a largely homogeneous group, potentially conflating ancient shared drift with a more recent, structured gene flow [28, 34, 39, 40, 41, 42]. Second, existing work has primarily compared populations across elevation gradients to identify adaptive loci, without systematically addressing the ancestral sources from which adaptive variants originate, the historical routes through which they entered the plateau, or the temporal sequence of these contributions [28, 34]. An integrated framework connecting who contributed to ancestry (sources and routes), when contributions occurred (temporal layering), and how these layers map to complementary physiological functions is therefore lacking. Addressing these gaps requires a synthesis of frequency‐ and haplotype‐based inferences, spatial diffusion models, and route‐specific scans.

Here, we assembled whole‐genome data from 1054 chickens spanning 11 Tibetan populations and 55 geographically adjacent reference populations to move beyond single‐method, single‐timescale approaches that have characterized previous studies of Tibetan chicken adaptation [28, 34, 39]. By integrating complementary admixture modeling, local‐ancestry inference, spatial gene‐flow estimation, and selection scanning approaches, we aimed to (i) resolve the number and identity of ancestral sources contributing to Tibetan chickens, (ii) determine the temporal hierarchy of their contributions, and (iii) assess whether each source is associated with distinct adaptive functions. This source × time × function framework offers a more integrative perspective on how a domestic species, shaped jointly by environmental pressure and human‐mediated dispersal, assembled its genetic architecture for life on the world's highest plateau.

## 2 Results

### 2.1 Sampling Design and Population Genetic Structure

**FIGURE 1:**
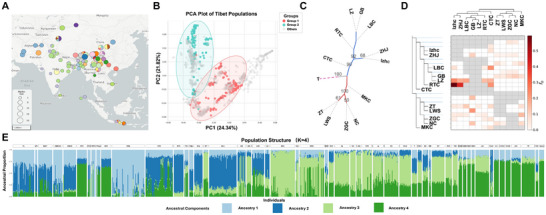
Sampling design and genetic structure of Tibetan chickens and neighboring domestic chicken populations. (A) Sampling map. Each site is shown as a circle whose radius is proportional to sample size; pie‐chart colors indicate the composition of populations/geographic blocks at that site. *n*‐values for each population are listed in Table S1. (B) Global principal component analysis (PCA). Individuals from non‐Tibet blocks are shown in gray; colored points are from the Tibet block. Two ellipses highlight the Sichuan–Yunnan Adjacent (SYA) lineage and the Southern Himalayan Foothills (SHF) lineage. PC1 and PC2 explain 24.34% and 21.82% of the total variance, respectively. (C) Population‐level unrooted ML tree of Tibetan and neighboring populations (13 groups). Branch colors distinguish the SHF‐proximal lineage (blue: CTC, lzhc, ZHJ, RTC, LBC, GB, and LZ) from the SYA‐proximal lineage (red: MKC, ZGC, NC, LWS, and ZT); T (outgroup) in pink. Numbers at internal nodes indicate Felsenstein bootstrap proportions (100 rapid bootstrap replicates on a 10% site subsample; see Methods 4.3.1). Only values ≥50 are shown. (D) Within‐Tibet F‐branch heatmap (Dsuite v0.5) [44]. Color intensity denotes the F‐branch value, which estimates the fraction of the genome affected by branch‐specific introgression. Colored (non‐gray) cells passed the default significance threshold (*p* < 0.01, block‐jackknife with *k *= 20 genomic blocks); f4‐ratio trios with *p* > 0.01 were set to zero before min–median aggregation. Of the 55 non‐zero cells, 16 are strongly supported (*Zb* > 3, approximately *p* < 0.003), including the strongest signals within the SHF lineage (RTC → lzhc, *fb* = 0.60, *Zb* = 23.8; LZ → lzhc, *fb* = 0.24, *Zb *= 10.6; LZ → LBC, *fb* = 0.19, *Zb *= 17.6) and within the SYA lineage (NC → MKC, *fb* = 0.09, *Zb *= 6.3; GB → MKC, *fb* = 0.07, *Zb* = 3.4). Cross‐lineage cells are uniformly weaker and largely non‐significant. (E) Global ADMIXTURE results (*K* = 4; cross‐validation error minimized at *K* = 15, see Figure S2). Each individual is a vertical bar with colors representing ancestry components. Panel A was generated using Microreact [45] (https://microreact.org/); basemap: Maps Mapbox (www.mapbox.com/about/maps) and OpenStreetMap (www.openstreetmap.org/about). Statistical panels were generated in R or Python; composite layout and annotation were performed in Microsoft PowerPoint (Microsoft 365).

This study analyzed whole‐genome data from 1054 chickens [43] to investigate the genetic diversity and adaptation of Tibetan chickens. The dataset comprised 322 Tibetan chickens (11 populations) and 732 reference samples from 55 geographically adjacent populations (Figure 1A; Table S1). Sample sizes ranged from 8 (GH) to 62 (LZ) individuals per Tibetan population; a summary of population‐level sample sizes and their inclusion in each downstream analysis is provided in Table S1B. All samples achieved a median sequencing depth of at least 10×, yielding approximately 90.7 million raw biallelic variant sites with a reliable Ts/Tv ratio of 1.69.

To elucidate the genetic structure of the study populations, we grouped all samples into nine geographic blocks: Tibet, Yunnan, Sichuan, Gansu–Shaanxi, Qinghai, Xinjiang, Bangladesh, India–Pakistan, and Central Asia (Figure S1A). Subsequently, we performed principal component analysis (PCA) on these blocks (Figure S1B). The results indicated that the Tibetan chicken populations within the Tibet block diverged into two primary genetic lineages (Figure 1B, PC1 and PC2 explained 24.34% and 21.82% of the total variance). These two lineages corresponded with their geographical distributions: one cluster comprised Tibetan chicken populations from areas adjacent to the Sichuan–Yunnan Adjacent lineage (SYA), whereas the other cluster consisted of populations from the infiltration zone near the Southern Himalayan Foothills lineage (SHF) (Figure S1C).

This two‐lineage pattern is corroborated by maximum‐likelihood (ML) phylogenies and F‐branch analyses (Figure 1C,D). Non‐zero F‐branch values passed the default significance threshold (*p* < 0.01, block‐jackknife, *k* = 20), with a strongly supported subset (16 of 55 cells; *Zb* > 3, approximately *p* < 0.003) concentrated within lineages gene‐flow signals (e.g., RTC–lzhc *fb* = 0.60, *Zb* = 23.8; LZ–LBC *fb* = 0.19, *Zb *= 17.6) but markedly weaker or non‐significant cross‐lineage exchange.

The PCA‐identified two‐lineage split (Figure 1B) was corroborated by ADMIXTURE analysis (*K* = 4; Figure 1E) [46]. Although the cross‐validation error decreases monotonically and reaches its minimum at *K* = 15 (Figure s2 A), *K* = 4 was selected for display because it most clearly resolves the SYA–SHF split [47]; higher *K*‐values further subdivide reference populations but do not alter the two‐lineage pattern within TIB (Figure S2B). We therefore treated SYA and SHF as two working lineages for subsequent demographic and adaptation analyses.

The consistent SYA–SHF split across PCA, phylogeny, and ADMIXTURE supports two working lineages within TIB. Whether these reflect a two‐source or more complex admixture history is tested in the next section using formal admixture models.

### 2.2 A Three‐Source Admixture Model for Tibetan Chickens

To test whether TIB ancestry can be explained by admixture between just two sources, we first computed the f3 admixture statistic. f3(TIB; SYA, SHF) was strongly negative (*f3* = −0.0019, *Z* = −33.1; Table S2), rejecting the null hypothesis that TIB derives from a simple tree involving only SYA and SHF. This result prompted us to re‐examine the PCA, where we noted that NWC occupies an intermediate position between TIB and other source populations in PC space (Section 2.1; Figure 1B), consistent with its contribution of a distinct ancestry component to TIB. This pattern was further corroborated by ADMIXTURE analysis, in which a NWC‐associated component was consistently identified at *K* = 3–5 (Figure 1E). Together, these lines of evidence motivated the inclusion of NWC as a candidate third ancestry source.

NWC is required as a third source. f4(NWC, SYA; TIB, Outgroup) was significantly negative across outgroup configurations (*Z* = −2.1 to −14.2; Figure S3A; Table S3), indicating excess allele sharing between NWC and TIB beyond what any tree topology would produce. All three two‐source models (SYA+SHF, SYA+NWC, SHF+NWC) were rejected by qpAdm under outgroup sets with adequate resolution (*p* < 0.01; Table S4). When we transferred NWC from the source set to the outgroup set, the remaining SYA+SHF model was rejected more strongly still, indicating that NWC contributes variation to TIB that neither SYA nor SHF can account for.

**FIGURE 2:**
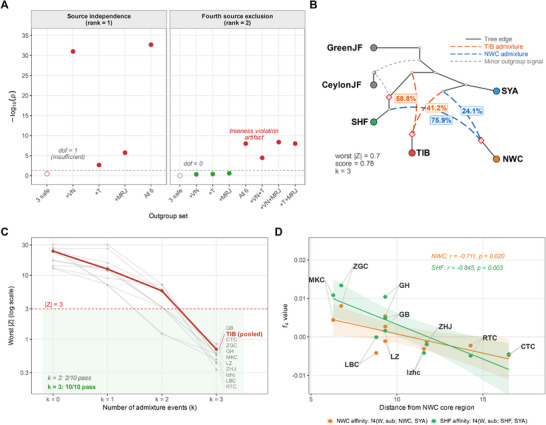
Establishing and validating a three‐source admixture model for Tibetan chickens (TIB) using qpWave rank tests and qpGraph topology modeling. (A) qpWave rank test results under nested outgroup configurations. Left panel: rank = 1 tests for {SYA, SHF, NWC}. Right panel: rank = 2 tests for {SYA, SHF, NWC, TIB}. All *p‐*values computed via block‐jackknife with 5 Mb blocks (admixtools2 default). (B) Best‐fit qpGraph topology for pooled TIB (*n* = 309) at *k* = 3 admixture events (worst |*Z*| = 0.70). TIB received 58.8% of its ancestry from a lineage shared with SHF and NWC, and 41.2% from a SYA‐related lineage. NWC is modeled as an admixed node (75.9% SHF‐related, 24.1% SYA‐related). Admixture weights are point estimates from the best‐fit qpGraph topology; cross‐method concordance (ADMIXTURE *K* = 4: ∼60% non‐SYA; Flare LAI: 30.3% combined SHF + NWC) supports the approximate magnitude. The *k* = 3 topology was required for all 10 subpopulations (worst |*Z*| = 0.33–0.84); block‐jackknife 95% CI for individual weights are wide (e.g., TIB SHF/NWC‐related: 50.9%–96.5%) owing to correlated estimation across genome blocks, but the topological requirement for three admixture events is invariant. (C) Per‐subpopulation qpGraph model adequacy. Each point represents the worst residual |*Z*|‐score for the best‐fit model at a given admixture complexity (*k *= 0–3). Dashed line: |*Z*| = 3 acceptance threshold. (D) Geographic gradient of NWC affinity across TIB subpopulations. f4(W, subpop; NWC, SYA) correlates positively with longitude (*r* = 0.711, *p* = 0.021, two‐sided). Error bars: ±1 SE from block‐jackknife. Shaded bands: 95% CI of linear regression. Statistical panels were generated in R or Python; composite layout and annotation were performed using the cowplot R package.

Having established that a three‐source model is necessary, we used qpWave [48, 49] rank tests to formally evaluate the dimensionality of the ancestry space (Figure 2A). With three phylogenetically safe outgroups (Green Junglefowl, Grey Junglefowl, and Ceylon Junglefowl), rank = 0 was rejected (*p* = 3.9 × 10^−38^; Table S5), meaning the three source groups are not interchangeable. Under expanded outgroup configurations (see Methods 4.4.3), each addition caused rejection of rank = 1: VN (*p* = 1 × 10^−31^), T (*p* = 2.2 × 10^−3^), and MRJ (*p *= 1.9 × 10^−6^). SYA, SHF, and NWC therefore represent three irreducible ancestry streams, robust to outgroup choice.

We next further tested whether a fourth component is needed. In all four outgroup configurations with adequate degrees of freedom, the rank = 2 test for {SYA, SHF, NWC, TIB} was accepted (*p* = 0.256 to 0.465): TIB lies within the three‐dimensional ancestry space of these three sources. To ask whether any plausible additional lineage contributes ancestry not captured by SYA, SHF, and NWC, we individually appended three candidate populations—White Leghorn (a globally distributed commercial breed), Red Junglefowl T (the wild progenitor present in the sampling region), and Chabo (a Japanese bantam breed of Southeast Asian origin, representing an independent East Asian domestication lineage) [50]—to the source set and repeated the rank test. In each case, rank = 2 was still accepted (*p* = 0.420, 0.164, and 0.262, respectively), indicating that none contributes an independent ancestry dimension beyond the existing three sources. When two or more of these populations served as outgroups simultaneously, rank = 2 was nominally rejected; however, treeness tests attributed these rejections to outgroup assumption violations rather than genuine additional ancestry (Figure S3B–D; Table S7).

To evaluate whether the three‐source definitions might mask finer substructure, we systematically tested within‐source splits. Splitting SYA into core Yunnan (SJ) versus Sichuan‐transition (XS) populations, or NWC into Gansu–Qinghai core (TP) versus peripheral (BYC) populations, did not increase the rank of the ancestry space (rank = 2 accepted; *p* = 0.225–0.491; Table S6). Splitting SHF into Pakistani (BJST) and Indian (INDIA) sub‐lineages indicated an additional statistical dimension (rank = 2 rejected; *p* = 0.004–0.011) indicating that these two sub‐lineages contribute partially distinguishable ancestries to TIB. Despite this added dimensionality, qpAdm replacement tests showed that the composite SHF definition remains an adequate proxy for downstream modeling, with TIB's South Asian ancestry predominantly affiliated with the Indian sub‐lineage (Table S8).

The qpWave analyses validated the three‐source framework, but qpAdm consistently returned negative weights for NWC (−0.15 to −1.32; Table S4), suggesting that NWC does not act as a simple, direct source but instead contributes to TIB through a more complex admixture graph that a linear mixture model cannot capture. We therefore turned to qpGraph [48, 49] with automated topology search (see Methods 4.4.4) to resolve the population relationships directly. Because qpGraph is sensitive to within‐source heterogeneity, we constructed pure‐source proxies (SHF_pure, NWC_pure, SYA_pure) by ranking individuals within each source group by their source‐diagnostic f4 affinity and retaining the top 15 per group (Methods 4.4.4).

To determine the minimum model complexity required to fit the data, we tested qpGraph models with *k* = 0 to *k *= 3 admixture events using six populations (GreenJF, GreyJF/CeylonJF, SHF_pure, NWC_pure, SYA_pure, and one TIB subpopulation; Figure 2C). Models with *k *= 2 were adequate for only 2 of 10 TIB subpopulations (worst |*Z*| ≥ 3.19 for the remaining 8), confirming that a two‐event history is insufficient for most of the plateau. By contrast, models with *k* = 3 fit well for all 10 subpopulations (worst |*Z*| = 0.33 to 0.84; Table S9), establishing three admixture events as both necessary and sufficient to explain the observed f‐statistic residuals. Pooling all TIB samples (*n* = 309), the best *k* = 3 graph (worst |*Z*| = 0.70; Figure 2B) attributed 58.8% of TIB ancestry to SHF/NWC‐related lineages and 41.2% to SYA‐related lineages, concordant with ADMIXTURE K = 4 (∼60% non‐SYA components) and Flare LAI (30.3% combined SHF + NWC). Notably, across all subpopulation‐specific graphs, NWC was consistently modeled as an admixed node receiving 53%–94% from a SHF‐related lineage, with the remainder from SYA‐related or deep ancestral lineages (Figure S4). This topology resolves the negative qpAdm weights observed above: NWC is not an independent source lineage but itself a product of earlier admixture, so a linear three‐way model necessarily assigns it a negative coefficient. Nonetheless, removing NWC from the graph degraded the fit for 8 of 10 subpopulations (worst |*Z*| ≥ 3.19), confirming that all three ancestry streams remain necessary.

To provide independent spatial validation of the three‐source model, we examined the geographic distribution of ancestry components across TIB subpopulations (Figure 2D). NWC affinity decreased significantly with distance from the Gansu–Qinghai region (*r* = −0.717, *p* = 0.020), consistent with diffusion via the Qinghai–Tibet corridor. A longitudinal cline was also evident when NWC and SHF were contrasted directly: f4(W, subpopulation; NWC, SHF) correlated strongly with longitude (*r *= −0.845, *p* = 0.002; Figure 2D), indicating that eastern subpopulations carry proportionally more NWC ancestry while western subpopulations are more SHF‐affiliated. The same spatial analysis also revealed distinct geographic signatures for the other two sources: SHF affinity was highest near the Yunnan–Myanmar border, whereas SYA did not follow a simple distance‐decay pattern but was concentrated at nodes in the Lhasa–Shigatse region.

### 2.3 Geographic Corridors and Barriers to Gene Flow

**FIGURE 3:**
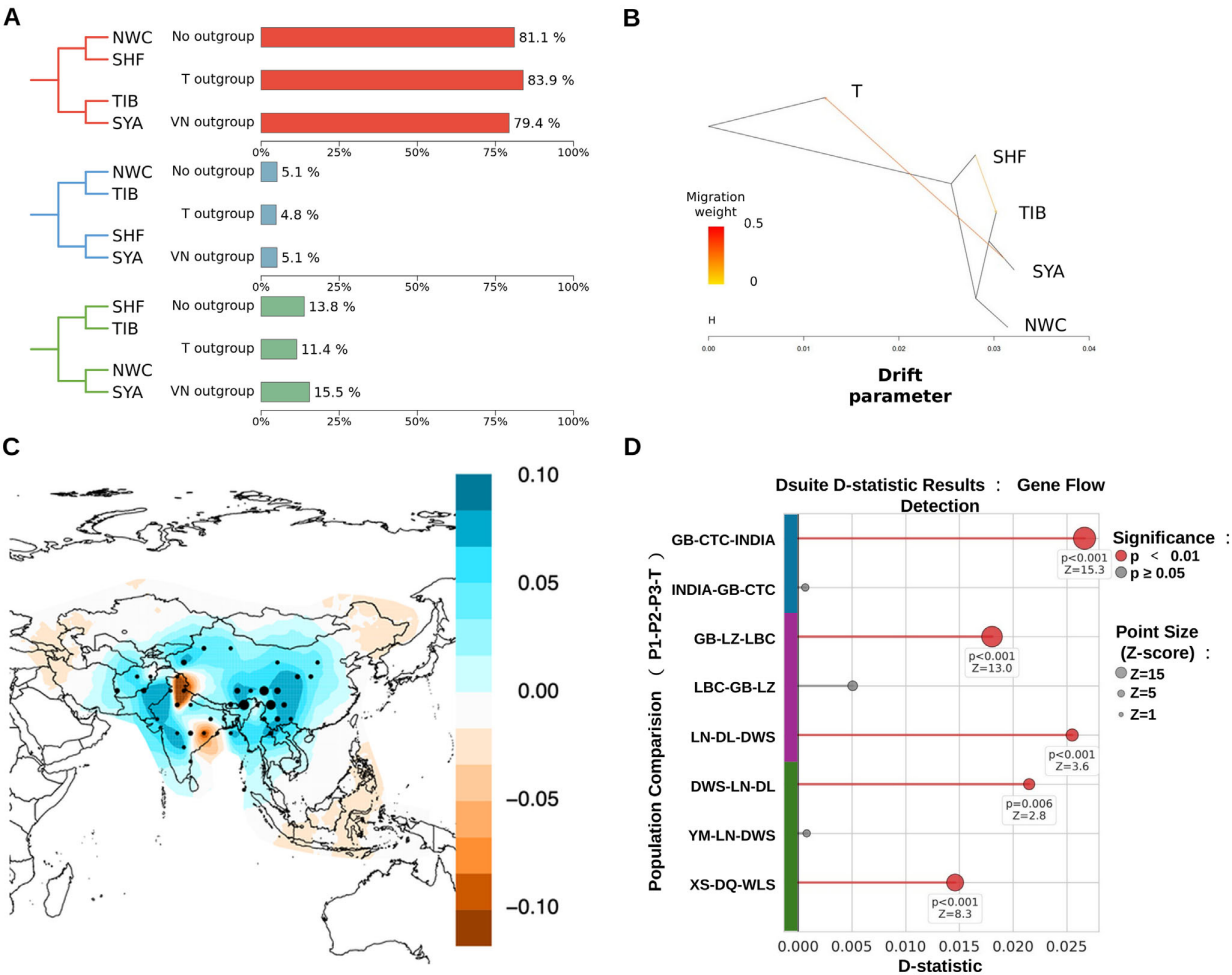
Phylogenetic structure and geographic corridors shaping gene flow in Tibetan chickens. (A) Genome‐wide topology weights (consensus‐sequence + PhyML BIONJ; 50‐SNP sliding windows) for four populations (TIB, SYA, SHF, and NWC) under three possible unrooted topologies and three outgroup conditions. ((TIB, SYA), (SHF, NWC)) is the dominant topology across all conditions: 81.1% [95% CI: 80.4%–81.9%] (no outgroup, *n* = 11 243 windows), 83.9% [83.3%–84.4%] (T outgroup, *n* = 17 369), 79.4% [78.8%–80%] (VN outgroup, *n* = 17 201). 95% CIs from 10 000 window‐level bootstrap resamples. Per‐chromosome breakdown in Figure S5A,B. (B) TreeMix population graph (T outgroup, *m* = 2). Migration arrows: SHF→TIB (*w* = 0.191 ± 0.005) and SYA→T (*w* = 0.348 ± 0.036; modeling artifact, see text). Standard errors estimated by block bootstrap (−*k* 1000). VN‐outgroup analysis (*m* = 3) shown in Figure S5F. (C) Estimated Effective Migration Surfaces (EEMS). Warm colors: below‐average effective migration (barriers); cool colors: above‐average (corridors). Black dots: observed demes (*n* = 982 individuals, 81 locations, 1 123 489 SNPs). (D) Four‐population D‐statistics (Dsuite). Colored bands group tests by region. Red points: significant gene‐flow signals (*p *< 0.01); point size scales with |Z|‐score. Statistical panels were generated in R or Python; composite layout and annotation were performed in Microsoft PowerPoint (Microsoft 365).

The three‐source model identifies the ancestral components of TIB but does not specify their phylogenetic relationships or geographic routes of entry. To examine phylogenetic structure, we combined topology weighting (Twisst [51]) with graph modeling (Treemix [52]) and F‐statistics. Twisst [51] identified ((TIB, SYA), (SHF, NWC)) as the dominant unrooted topology (81.1% of 11 243 genomic windows, 95% CI: 80.4%–81.9%; Figure 3A; Figure S5A,B), placing TIB as a sister to SYA across most of the genome. Treemix with the optimal number of migration edges (*m* = 2) and Red Junglefowl (T) as outgroup recapitulated this backbone and inferred SHF→TIB gene flow (*w* = 0.191 ± 0.005), alongside an SYA→T edge (*w* = 0.348 ± 0.036; Figure 3B). However, independent f_4_ and F‐branch tests actively reject excess SYA–T affinity, indicating that this edge most likely represents a modeling artifact rather than genuine gene flow (Figure S5C–E): SYA‐centered f_4_ contrasts—f_4_(SYA, B; C, T) with B, C ∈ {TIB, SHF, NWC}—showed all significant |Z|‐scores confined to ingroup permutations and no excess SYA–T affinity, and F‐branch clustering grouped SYA with TIB and away from T (Figure S5C–E). Using an alternative outgroup (Vietnamese populations, VN) with the optimal number of edges (*m* = 3) yielded an equivalent ML backbone (Figure S5F). Together, these results support an SYA–TIB sister relationship with additional SHF→TIB introgression, consistent with NWC deriving from admixture of SHF‐related and SYA‐related lineages (Section 2.2), with its temporal depth resolved in Section 2.4.

To map the spatial channels through which the three ancestries reached the plateau, we estimated effective migration surfaces and validated them with four‐population D‐statistics. The Estimated Effective Migration Surface (EEMS) analysis [53, 54, 55] (Figure 3C) identified three significant geographical features: (i) the main ridge of the Himalayas corresponds to a low‐migration zone; (ii) the Yarlung Tsangpo Grand Canyon coincides with a high‐migration corridor; and (iii) the Yunnan–Guizhou Plateau presents a broad surface of moderate to high migration with dispersed patches of low migration at the edge of the Hengduan Mountains. Because EEMS surfaces reflect cumulative effective migration rates and are sensitive to both sampling density and geographic coverage gaps, the inferred corridors represent probabilistic zones of elevated gene flow rather than precise historical pathways.

Patterson's D‐statistics supported the EEMS‐inferred corridors [44] (Figure 3D; Table S10). Across the Himalayan main ridge, the GB–CTC–INDIA comparison was significantly positive (*D* = 0.0267, *Z* = 15.28, *p* < 1 × 10^−^^1^ ^5^), supporting the ridge as a barrier. Around the Yarlung Tsangpo Grand Canyon, GB–LZ–LBC was positive (*D* = 0.0180, *Z* = 12.98, *p* < 1 × 10^−^^1^ ^5^), indicating a high‐migration corridor. On the Yunnan–Guizhou Plateau, both directions along LN–DL–DWS were positive (*D* ≈ 0.02–0.03), consistent with broad connectivity, whereas contrasts involving the Hengduan fringe were nonsignificant, revealing fine‐scale topographic barriers.

Having established the phylogenetic backbone and spatial corridors linking the three ancestries to TIB, we next resolved the temporal sequence of these inputs using local‐ancestry inference and tract‐length dating.

### 2.4 Local Ancestry Architecture and Admixture Dating

#### 2.4.1 Three‐Ancestry Mosaic and Geographic Gradients

To resolve both the chronology and geography of the Tibetan chicken formation, we integrated local‐ancestry inference (LAI) with tract‐length dating [56], separating recent pulses from older ancestry layers.

**FIGURE 4:**
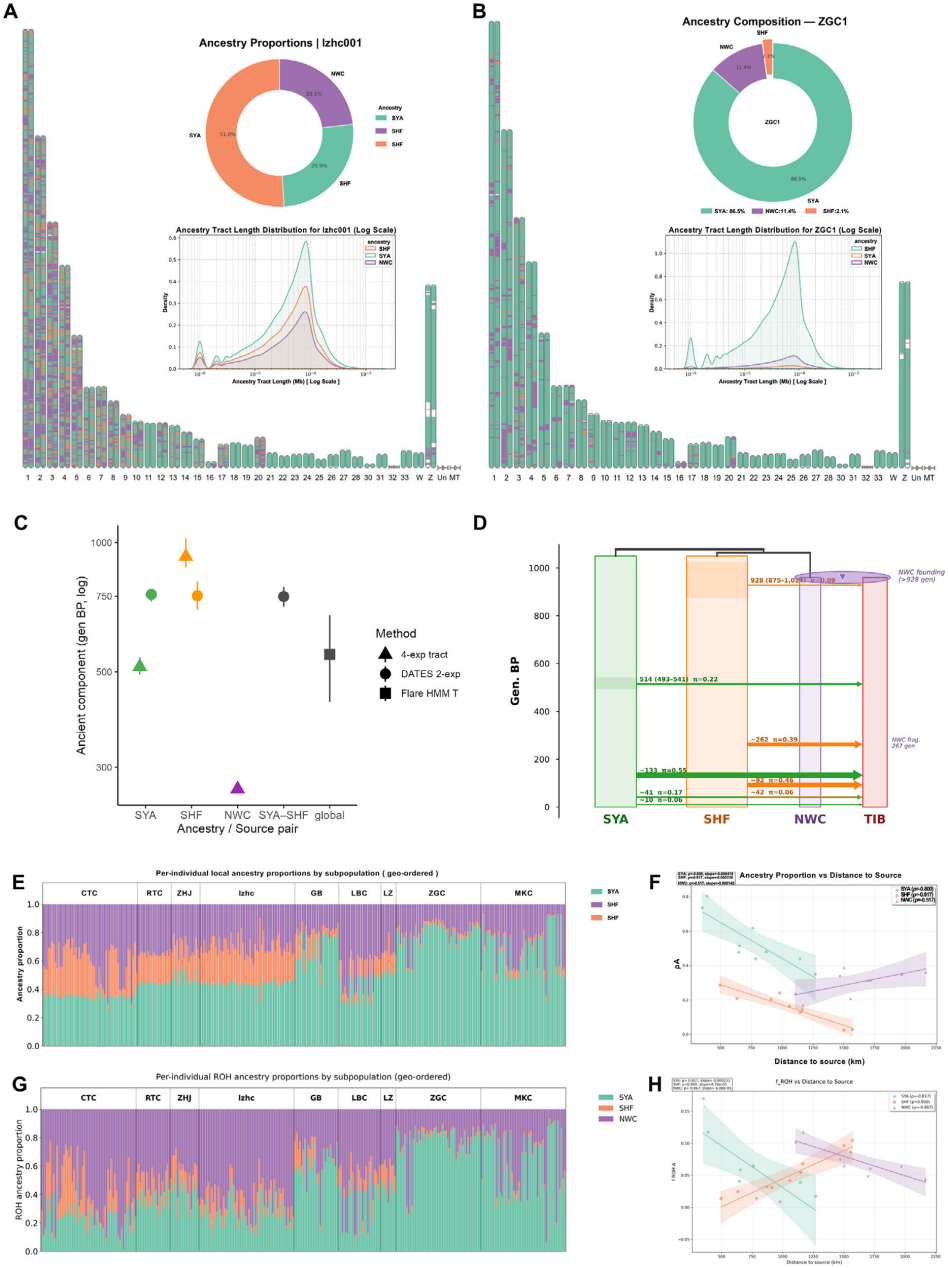
Local ancestry architecture, admixture dating, and temporal model of Tibetan chickens (TIB). (A,B) Local ancestry for two representative TIB individuals. Each panel shows a donut chart of genome‐wide ancestry proportions (purple: NWC; orange: SHF; green: SYA), chromosome‐level ancestry painting (rows: autosomes chr1–33; color: assigned ancestry as in the donut chart), and an inset density plot of ancestry tract lengths (log_10_ scale). (A) lzhc001 from Longzi County (SYA 51%, NWC 23.1%, SHF 25.9%). (B) ZGC1 from Zuogong County (SYA 86.5%, NWC 11.4%, SHF 2.1%). (C) Cross‐method comparison of ancient admixture time estimates. Points: median ancient‐component estimate; whiskers: 95% CIs from parametric bootstrap (4‐exp tract), nonparametric bootstrap (DATES 2‐exp, target = TIB), or chromosome‐level bootstrap (Flare HMM T). DATES source pairs are mapped to the ancestry whose influx they most directly estimate: NWC–SYA → SYA, NWC–SHF → SHF; the SYA–SHF pair is shown separately as a cross‐check (*n* = 284–292 per pair after QC). (D) Schematic admixture model integrating the four‐tier temporal framework. Tree topology from qpGraph (*k* = 3; Figure 2B); sample sizes from Table S1. Left axis: generations before present. Colored horizontal arrows represent admixture pulses from SYA (green) and SHF (orange) into TIB at four time depths; arrow thickness is proportional to mixture weight (π) from the 4‐component tract‐length model (Table 1; Table S12). Purple shaded band: NWC ancestry layer, whose onset (>928 generations) is constrained by the SHF ancient component; dotted line at ∼267 generations marks the most recent large‐scale fragmentation of NWC tracts. Brackets on ancient‐layer arrows: 95% CIs from parametric bootstrap (SYA: 493–541 gen; SHF: 875–1024 gen). Gray annotations: cross‐method validation from DATES 2‐exponential fits and Flare HMM transition‐rate parameter (Table S12). (E) Per‐individual local ancestry proportions ordered geographically by subpopulation (west to east). (F) Ancestry proportion versus geographic distance (9 TIB subpopulation medians). For SYA and SHF, distance is measured from each ancestry's own source center; for NWC (resident ancestry layer), distance is measured from the SYA/SHF centers, so that the positive correlation reflects NWC enrichment in areas far from the two introgressing sources. Spearman rank correlations (two‐sided): SYA *ρ* = −0.73 (*p* = 0.025), SHF *ρ* = −0.87 (*p* = 0.003), NWC *ρ* = +0.65 (*p* = 0.058, marginally significant). Lines: LOESS regression; shaded bands: 95% confidence intervals. (G) Per‐individual ROH ancestry composition by subpopulation, ordered as in (E). Each ROH segment is classified by the predominant local ancestry. Per‐subpopulation mean ± 95% bootstrap CI (10 000 resamples): CTC (*n* = 56) SYA 23.2% [20.7–25.7], NWC 59.6% [54–65.2]; RTC (*n* = 20) SYA 36.5% [33.5–39.5], NWC 50.5% [46.8–54.3]; ZHJ (*n* = 17) SYA 41.1% [36.6–45.4], NWC 45.3% [40.1–51]; lzhc (*n* = 56) SYA 26.1% [23.8–28.5], NWC 63.1% [60.1–66]; GH (*n* = 9) SYA 67.6% [64–71.1], NWC 31.4% [27.6–35.1]; LBC (*n* = 25) SYA 34.6% [28.3–40.6], NWC 55.8% [48.2–63.9]; LZ (*n* = 9) SYA 44.2% [40.6–47.5], NWC 42.3% [37.6–49]; MKC (*n* = 50) SYA 64.2% [57.8–70.4], NWC 33.5% [27.6–40]; ZGC (*n* = 50) SYA 76.3% [72.2–79.6], NWC 21.6% [18.2–25.7]; GB (*n* = 17) SYA 56.2% [49.3–62.1], NWC 33.1% [26–41.6]. (H) Ancestry‐specific ROH frequency (f_ROH) versus geographic distance from source centers (9 subpopulation medians). Spearman rank correlations (two‐sided): SYA *ρ* = −0.82 (*p* = 0.007), NWC *ρ* = −0.87 (*p* = 0.003), SHF *ρ* = +0.90 (*p* = 0.001). Format as in (F). Statistical panels were generated in R or Python; composite layout and annotation were performed in Microsoft PowerPoint (Microsoft 365).

We applied Flare [57] for LAI using SYA, SHF, and NWC as reference panels. Chromosome painting showed a three‐tier mosaic architecture across all TIB individuals: short, highly fragmented SHF tracts (mean 0.24 Mb), moderately intact NWC segments (0.53 Mb), and long, contiguous SYA blocks (0.65 Mb), with the relative proportions varying predictably by geographic proximity to each source center (Figure 4A,B,E; Figure S6A,C). Genome‐wide, LAI assigned on average 54.7% of TIB haplotype‐sites to SYA, 28.1% to NWC, and 17.1% to SHF (soft‐call posterior averages across 309 individuals; hard‐call proportions were similar: 56.1%, 27%, 16.9%). Because LAI relies on haplotype‐pattern matching, it is inherently most sensitive to long, recently introduced tracts; ancient tracts fragmented by recombination below the effective detection resolution are systematically underassigned. Validation via pseudo‐admixed and leave‐one‐out benchmarks confirmed per‐site accuracy of ∼88%–90%, with the dominant error being SYA→NWC misassignment (15.2%; Figure S7B,C,E), consistent with this expected bias. Since the bias direction—SYA overestimated, NWC and SHF underestimated—is opposite to what would undermine the three‐tier temporal model, the LAI‐based portrait represents a conservative estimate of the older layers' true contributions.

Correlating subpopulation‐level ancestry proportions with distance from each source center revealed consistent spatial gradients (Spearman rank correlation across 9 subpopulation medians): SYA decreased with distance from its source center (*ρ* = −0.73, *p* = 0.025), SHF decayed sharply from the southern source (*ρ* = −0.87, *p* = 0.003), and NWC showed the complementary pattern (*ρ* = +0.65, *p* = 0.058; Figure 4F). The three gradients thus form a mirror‐image spatial structure—SYA and SHF declining, NWC increasing toward the plateau interior—whose dispersal implications are considered in Discussion 3.3.

#### 2.4.2 Multi‐Wave Admixture Dating

The systematic tract‐length differences across the three ancestries (Section 2.4.1) suggest temporally stratified admixture events. To test this independently, we applied DATES [58, 59] across three pairwise source combinations (NWC–SYA, SHF–NWC, SYA–SHF; see Methods 4.7.1). The ancestry‐weighted LD decay separated into an ancient component (median 749–757 generations across pairs; 95% CI: 698–811) and a recent component (37–45 generations; Figure S8A,B; Table S12), indicating that TIB experienced at least two temporally distinct admixture epochs. Flare's internal HMM transition‐rate parameter returned a genome‐wide median of ∼550 generations (95% CI: 427–677; Table S12), intermediate between the two DATES components.

To decompose these signals by ancestral source, we fitted k‐component truncated exponential mixture models to ancestry‐specific tract‐length distributions on macrochromosomes (chr 1–5; see Methods 4.7.2). BIC overwhelmingly favored the four‐component model for all three ancestries (Figure S8C). For external admixture sources (SYA, SHF), fragmentation time approximates entry time; for the resident founding population (NWC), it instead reflects when subsequent gene flow began breaking up resident haplotypes rather than when NWC ancestry first entered (see Discussion 3.1). The four‐component estimates are summarized in Table 1 (generation times converted at 1.5 years/gen).

**TABLE 1:** Four‐component tract‐length dating summary.

Ancestry	*t*_1_ (modern)	*t*_2_ (early‐int.)	*t*_3_ (late‐int.)	*t*_4_ (ancient)	*π*_4_
SYA	10 (9–10)	41 (38–44)	133 (128–139)	514 (493–541)	0.22
SHF	42 (40–45)	92 (90–94)	262 (255–272)	928 (875–1024)	0.09
NWC†	12 (12–13)	30 (29–31)	81 (76–85)	267 (262–272)	0.50

NWC^†^ is the resident founding substratum; its t_4_ dates the most recent large‐scale fragmentation event (the SYA influx), not the NWC founding time, which is constrained at >928 gen by the SHF ancient component.

Values in generations (95% CI from parametric bootstrap, 500 replicates). CIs reflect tract‐sampling variance only; see Methods 4.7.2 for additional uncertainty sources. *π* = mixture weight.

Four lines of evidence converge on a temporally ordered, multi‐wave admixture history. First, the SHF ancient component (928 generations; 95% CI: 875–1024) exceeded the DATES ancient estimates (749–757 gen; 95% CI: 698–811; Figure 4C), consistent with DATES capturing a composite signal across multiple waves rather than the deepest layer alone. Second, the NWC ancient component (267 gen) was notably younger than SYA (514) and SHF (928); this is expected for a resident ancestry whose tracts are dated by when subsequent introgression fragmented them, not by when NWC first entered TIB (Discussion 3.1). Consistent with this interpretation, merging NWC and SHF tracts into “non‐SYA” super‐tracts yielded an ancient component of 374 generations (95% CI: 360–389), aligning with the SYA pulse age (Figure S8D). Third, fastsimcoal2 [60] demographic modeling decisively favored episodic pulses over continuous migration (ΔAIC = 7212; Table S13); SFS‐based timing estimates were systematically deeper than tract‐length dates (NWC ∼ 3312, SHF ∼ 3126, SYA ∼ 1552 gen), consistent with the expected upward bias of coalescent methods (Methods 4.7.5), and are treated as order‐of‐magnitude upper bounds. The best‐fit Pulse model was used to parameterize neutral simulations for selection scans (Section 2.6). Finally, sensitivity analysis confirmed that the temporal ordering was invariant across recombination rates (1.2–3.11 cM/Mb; Figure S9A,B), tract‐length cutoffs, generation‐time assumptions, and per‐chromosome subsets (Figure S9C–E).

#### 2.4.3 Ancestry‐Stratified ROH Cross‐Validates the Temporal and Spatial Framework

The multi‐wave temporal model generates a testable prediction regarding runs of homozygosity (ROH): because recently introgressed ancestry tracts have had less time to be broken up by recombination, SYA—the most recent major pulse (∼514 gen)—should be enriched in long ROH, whereas the ancient, heavily fragmented NWC substratum should contribute preferentially to short ROH. Ancestry‐stratified ROH analysis confirmed this prediction: SYA ancestry increased monotonically from short ROH (∼57%) through mid‐length (∼65%) to long ROH (∼69%), whereas NWC decreased reciprocally (∼36% → 27% → 22%); SHF remained low and stable across all length classes (Figure S10).

The ROH signal further recapitulated the geographic ancestry gradients established by LAI (Section 2.4.1). Per‐subpopulation ROH composition mirrored the spatial structure, and ancestry‐specific ROH frequency decayed significantly with distance from each ancestry's source center (SYA *ρ* = −0.82, *p* = 0.007; NWC *ρ* = −0.87, *p* = 0.003; SHF *ρ* = +0.90, *p *= 0.001; Figure 4G,H). This independent cross‐validation, based on an entirely different genomic feature (autozygosity rather than haplotype assignment), strengthens confidence in the LAI‐based spatial portrait (Figure 4F; Discussion 3.3).

#### 2.4.4 Integrated Temporal Framework

Combining all dating approaches, the data support four resolvable time‐scales (Figure 4C,D; component estimates in Figure S8C; sensitivity in Figure S9): (i) a deep NWC ancestry layer whose founding predates the oldest tract‐length signal; the SHF ancient component (928 gen; 95% CI: 875–1024) and the DATES ancient estimates (749–757 gen; 95% CI: 698–811) provide independent lower bounds, placing NWC establishment at >928 generations (>1392 years BP); (ii) a major SYA influx at ∼514 generations (∼771 years BP; 95% CI: 493–541); (iii) an intermediate exchange epoch at 90–262 generations (∼135–393 years BP; 95% CI: 76–272); and (iv) a modern breed‐exchange signal at 10–42 generations (∼15–63 years BP; 95% CI: 9–45). This four‐tier architecture was robust across all sensitivity tests (Figure S9).

### 2.5 Fine‐Scale Diffusion Along Two Dispersal Routes

The four‐tier temporal framework (Section 2.4.4) resolves when each ancestry entered the Tibetan chicken gene pool, but not how it spread across the plateau—similar distance‐decay clines can arise from distinct dispersal processes. To characterize the route‐specific dynamics of the SYA and SHF corridors, we compared F‐branch gradients, IBD sharing, and route‐anchored regressions along each route (the NWC corridor was excluded owing to sparse spatial coverage; see Limitations).

**FIGURE 5:**
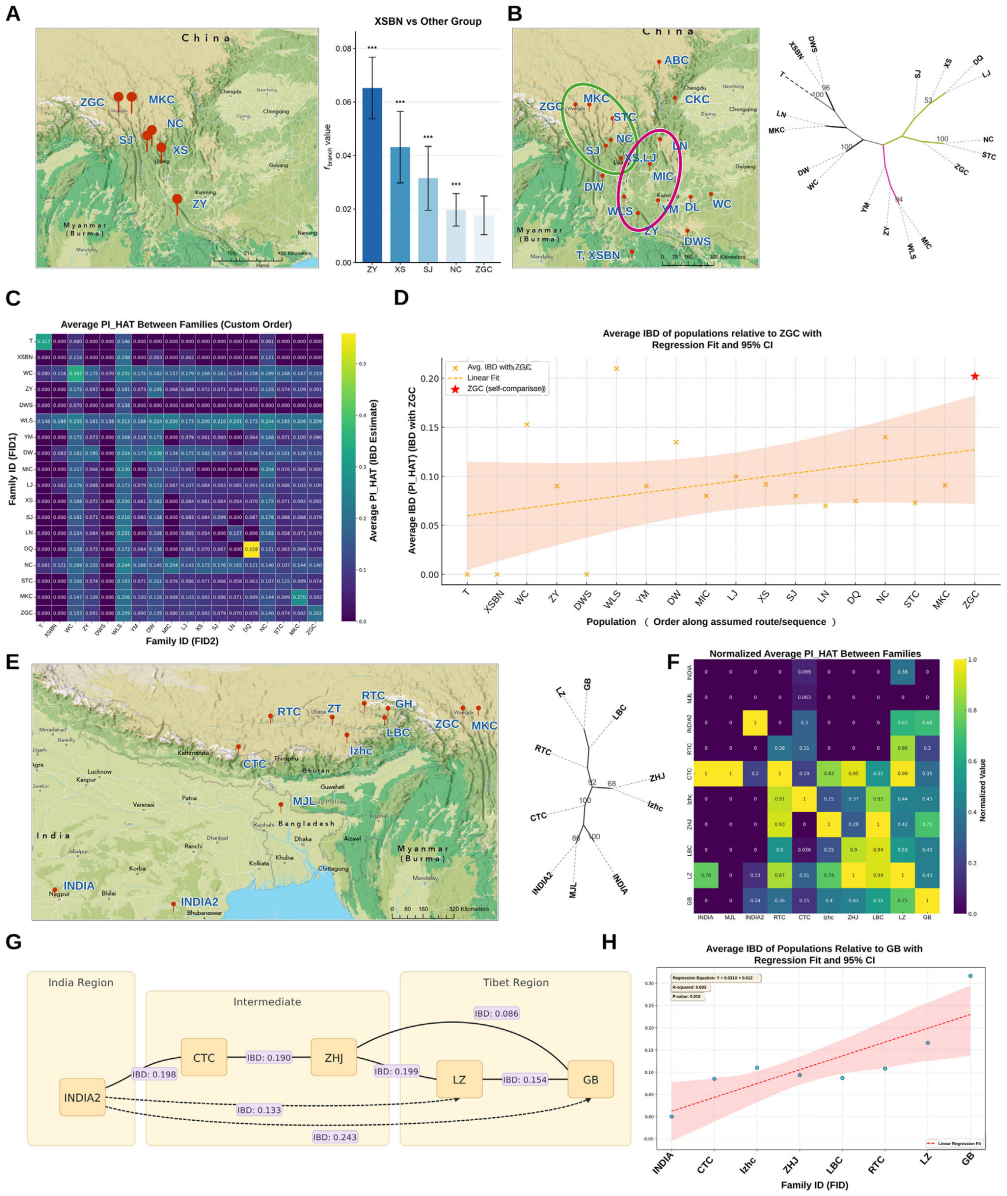
Fine‐scale genetic structure and diffusion patterns along two dispersal routes. (A–D) Southwestern (SYA‐proximal) route. (A) F‐branch values along the SYA corridor (Dsuite; error bars: chromosome‐jackknife SE). (B) Geographic microclusters and population‐level unrooted ML tree (18 groups; bootstrap values ≥ 50 shown at internal nodes; Methods 4.3.1). Branch colors: black (NWC‐affiliated), olive‐green (Yunnan SYA), magenta (Sichuan SYA). (C) Pairwise average PI_HAT heatmap along the SYA path (18 populations; sample sizes: T *n* = 10, XSBN *n* = 4, WC *n *= 8, ZY *n* = 26, DWS *n* = 3, WLS *n* = 23, YM *n *= 10, DW *n* = 9, MIC *n* = 13, LJ *n* = 21, XS *n* = 29, SJ *n* = 30, LN *n* = 28, DQ *n* = 10, NC *n* = 11, STC *n* = 20, MKC *n* = 48, ZGC *n* = 50). (D) Mean IBD (PI_HAT) with ZGC along the assumed route sequence (*n* = 14 populations); shaded band: 95% CI of linear regression (*r* ^2^ = 0.22, *p* = 0.088). Note that statistical power for route‐level regressions in (D,H) is limited by the small number of sampled populations along each corridor. (E–H) Southern Himalayan foothills (SHF) route. (E) Map and population‐level unrooted ML tree (10 groups; bootstrap values ≥ 50 shown at internal nodes; Methods 4.3.1). (F) Normalized pairwise average PI_HAT heatmap along the SHF path (10 populations; sample sizes: INDIA *n* = 17, MJL *n* = 4, INDIA2 *n* = 4, RTC *n* = 20, CTC *n* = 56, lzhc *n* = 56, ZHJ *n* = 17, LBC *n* = 24, LZ *n* = 9, GB *n* = 17). (G) Stepwise diffusion pathway along the SHF route; branch labels show pairwise mean IBD (PI_HAT) ± SE. (H) Mean IBD (PI_HAT) with GB along the SHF route (*n* = 6 TIB subpopulations); shaded band: 95% CI of linear regression (*r* ^2^ = 0.41, *p* = 0.17). The map panels in Figure 5 (panels A, B, and E) were produced in ArcGIS using its default elevation map as the basemap. Map image is the intellectual property of Esri and is used herein under license. Copyright 2026 Esri and its licensors. All rights reserved. Statistical panels were generated in R or Python; composite layout and annotation were performed in Microsoft PowerPoint (Microsoft 365).

The SYA corridor exhibited signatures of stepwise, population‐structured gene flow, with progressive attenuation of the admixture signal through a chain of intermediate populations. The F‐branch gradient declined monotonically from the source (XSBN: *fb *= 0.065 ± 0.012) toward increasingly distant plateau populations (NC: 0.020 ± 0.006; all *Zb* > 3), becoming non‐significant at the corridor terminus (ZGC: *fb* = 0.018 ± 0.007, *Zb* = 2.60; Figure 5A; Table S23). Despite this attenuation, IBD was elevated—rather than depleted—at downstream populations such as ZGC and MKC (Figure 5C), suggesting that repeated low‐level inflow compounds with local drift to produce terminal accumulation. Consistent with discrete population‐mediated relay rather than continuous diffusion, local phylogeny–geography mismatches revealed “neighboring‐but‐distinct” pairs that maintain genetic boundaries despite geographic proximity (Figure 5B). Route‐anchored regression using ZGC as a reference confirmed the overall distance‐dependent trend (Figure 5D).

By contrast, the SHF corridor displayed a smoother diffusion pattern with less evidence of discrete population‐mediated relay. The ML topology ordered populations in a nearest‐neighbor chain from South Asia into the plateau interior (India → CTC → ZHJ → LZ/GB; Figure 5E), and pairwise IBD declined along this path without the sharp inter‐population discontinuities observed on the SYA route (Figure 5F). CTC occupied a central position in IBD connectivity (Figure 5G), acting as a high‐throughput node rather than a discrete boundary. The route‐anchored regression with GB as a reference increased monotonically in the along‐corridor order (Figure 5H), consistent with distance‐mediated gene‐flow decay.

Taken together, the two corridors represent contrasting dispersal regimes—population‐structured relay along the SYA route versus more continuous diffusion along the SHF route—whose demographic implications are considered in Discussion 3.3. These route‐stratified patterns are subjected to demographic control and multi‐layer validation in the following section.

### 2.6 Route‐Stratified Selection Signals and Functional Enrichment

Given the complex demographic history of these populations (Sections 2.4–2.5), we applied a three‐layer validation framework—combining demographic calibration, pathway testing, and ROH control—to identify selection signatures that resist a purely demographic explanation (Methods 4.9–4.10).

#### 2.6.1 Demographic Calibration (Layer 1)

The complex three‐source admixture history of TIB can produce inter‐population divergence patterns that mimic signatures of positive selection; candidate identification, therefore, requires calibration against a demographic null. We used the best‐fit Pulse model (Section 2.4; Methods 4.10.1) to simulate a neutral genome‐wide null distribution via msprime [61, 62] and constructed a three‐component composite score—z(F_ST) + z(−π‐ratio) + z(XP‐EHH)—normalized against this null (Methods 4.10.2–4.10.3). The resulting null was conservative, with genomic inflation factors of 0.76–0.78 (Figure S12B–D), indicating that the calibration is unlikely to inflate false positives. Despite this stringency, the fraction of windows exceeding the neutral 1% tail remained approximately twofold above expectation across all three contrasts (2.17× for TIB vs. SYA, 1.99× for TIB vs. NWC, 2.13× for TIB vs. SHF; Figure S12E), confirming that a genuine selection signal persists after demographic correction. Windows reaching BH‐FDR *q* < 0.05 for any individual component statistic were retained as Layer 1 candidates (255–536 per contrast; Table S14; see Limitations for sensitivity to sweep architecture).

#### 2.6.2 Route‐Specific Functional Partitioning (Layer 2)

**FIGURE 6:**
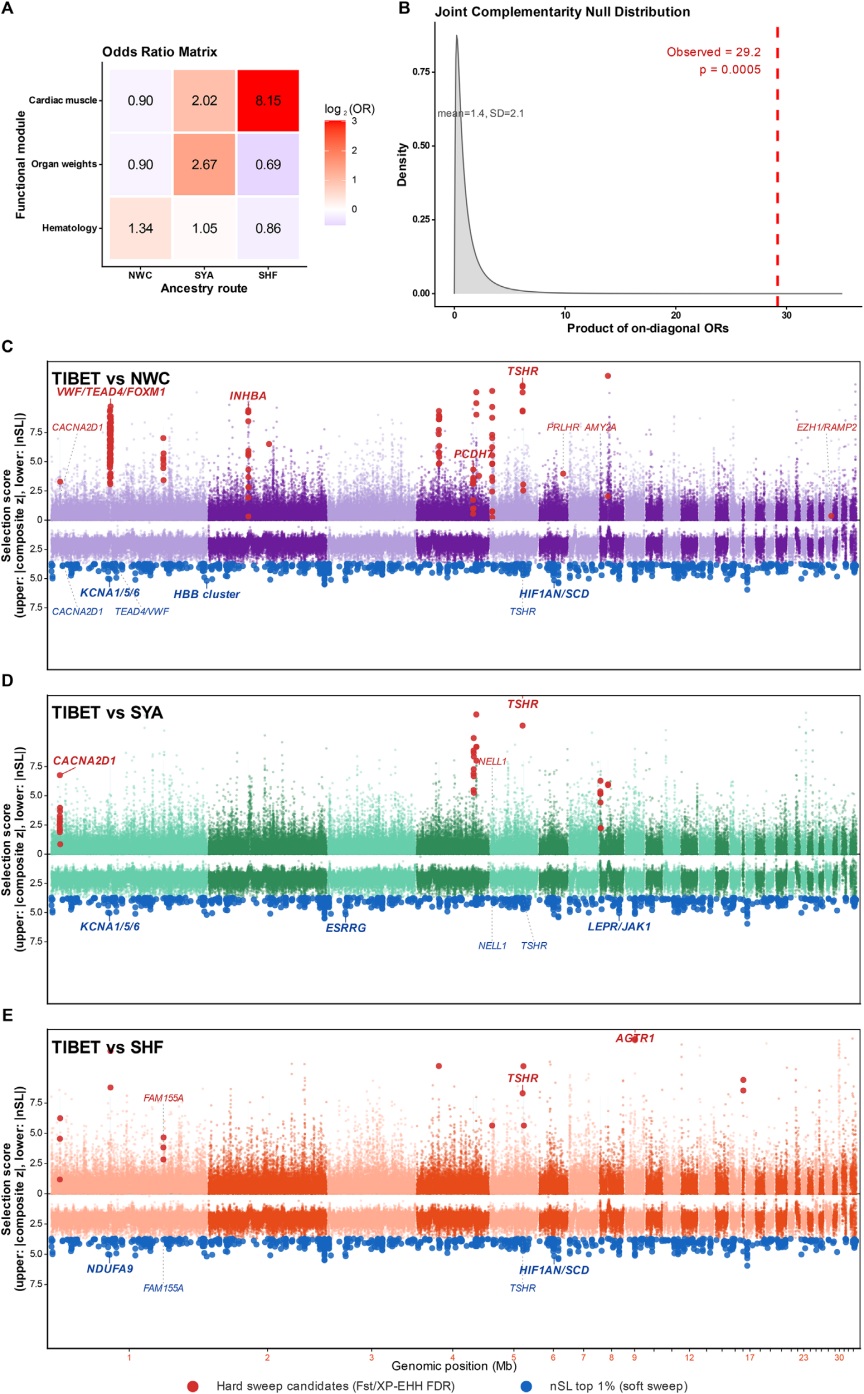
Three‐way functional complementarity and route‐stratified selection signals in Tibetan chickens. (A) 3 × 3 odds ratio matrix (3 ancestry routes × 3 functional categories). Color intensity scales with log_2_(OR). NWC‐route candidates are enriched for hematology‐related genes (*OR* = 1.34), SYA‐route for organ weight/growth (*OR* = 2.68), and SHF‐route for cardiac muscle contraction (*OR* = 8.15). (B) Permutation null distribution (10 000 iterations) of the diagonal OR product. Observed joint statistic = 29.19 (red dashed line) versus null mean = 1.4 (SD = 2.1); 99th percentile = 7.07; permutation *p* = 5 × 10^−^^4^, supporting the hypothesis of functionally complementary contributions from the three ancestral sources. (C–E) Mirror Manhattan plots of calibrated composite selection scores (upper half) and within‐population nSL scores (lower half) for TIB contrasted with NWC (C), SYA (D), and SHF (E). Upper panels: *n* = 96 095 non‐overlapping 10 kb windows on GRCg6a; the composite score combines z‐normalized F_ST, π‐ratio, and XP‐EHH, all calibrated against a neutral demographic null (λ_GC = 0.76–0.78; see Methods). Red dots: candidate windows exceeding Benjamini–Hochberg FDR *q* < 0.05 (Layer 1: NWC 536, SYA 255, SHF 437 windows). Lower panels: nSL (number of Segregating sites by Length) statistic computed within TIB using Selscan (v2.0), detecting ongoing or soft sweeps on standing variation. The 709 top‐1% nSL windows show near‐zero overlap (2–3 windows) with the 223 calibrated composite candidates, confirming that nSL captures a distinct evolutionary process. Notable nSL peaks include *KCNA1/KCNA5/KCNA6* (hypoxic pulmonary vasoconstriction; chr1), *NDUFA9* (mitochondrial complex I), *HIF1AN* (HIF‐1alpha inhibitor; chr6), *ESRRG* (mitochondrial biogenesis; chr3), and the *HBB* cluster. nSL has not been calibrated against the demographic null, and these candidates remain hypothesis‐generating. Statistical panels were generated in R or Python; composite layout and annotation were performed using the cowplot R package.

To investigate whether distinct ancestral sources provided specialized adaptive contributions, we evaluated the functional enrichment profiles of candidate genes associated with each ancestry route. Ancestry‐stratified enrichment testing indicated that the three ancestry routes harbor statistically distinguishable functional profiles (Methods 4.10.6): NWC‐route candidates were enriched for hematology‐related genes (*OR* = 1.34, *p* = 2.1 × 10^−^^4^), SYA‐route candidates for organ weight/growth genes (*OR* = 2.68, *p* = 6.2 × 10^−^^3^), and SHF‐route candidates for cardiac muscle contraction (*OR* = 8.15, *p* = 0.022; Figure 6A; Table S20). A joint permutation test indicated that this three‐way partitioning could not arise by chance (observed diagonal OR product = 29.19 vs. null 99th percentile = 7.07; permutation p = 5 × 10^−^^4^; Figure 6B; Table S19). We emphasize that this functional partitioning is inferred from statistical enrichment patterns rather than from experimental demonstration of phenotypic effects; the correspondence between ancestry route and biological function remains correlative.

#### 2.6.3 ROH Island Analysis (Layer 3)

While standard composite signals capture broad genomic differentiation, shared ROH islands provide distinct structural evidence of recent, intense selection. Therefore, true targets within these islands are expected to show enriched composite signals relative to the genomic baseline. Additionally, since Tibetan chickens have undergone both natural adaptation to high altitudes and artificial breeding for production, these ROH islands likely reflect a combination of both selective regimes.

Defining ROH islands at the 95th frequency percentile (Methods 4.10.7) yielded 58 regions. Intersecting these with our calibrated composite signals confirmed 13 true selection targets, which are overwhelmingly driven by NWC ancestry (*OR *= 10.91, *p* = 3.4 × 10^−^^1^ ^6^; Figure S14A,B).

Functional annotation of these 13 confirmed islands—based on gene ontology [63] and QTL database cross‐referencing [64] rather than direct experimental validation—is consistent with dual selective regimes acting on Tibetan chickens (Table S21). One class of genes is associated with altitude‐related functions, encompassing vascular coagulation (VWF, EFNB2), respiratory‐neural (NTF3, FOXM1), and metabolic/ion‐channel modules. The other class is associated with production traits, highlighted by NELL1 (skeletal development), NOBOX (egg production), and FKBP4/CD9 (reproductive function). The production‐trait signal is predominantly driven by body‐size selection, as evidenced by strong enrichment of body‐weight/growth QTLs within significant ROH islands relative to non‐significant ones (*OR *= 13.1, *FDR* = 7.6 × 10^−^^2^ ^2^; Figure S14C; Table S22d).

Notably, these two functional classes are not genomically segregated. At the dominant chr1: 74–75.7 Mb island (93 significant windows), the core vascular–respiratory adaptation cluster physically co‐occurs with production‐trait genes, including bridge loci such as TEAD4 (cardiac Hippo signaling and organ size) and CACNA2D1 (calcium signaling and body weight QTL) (Table S21). This tight co‐localization is more parsimoniously explained by linkage hitchhiking—in which selection on one functional class swept a shared haplotype carrying variants of the other class to high frequency—than by independent selection at each locus. The co‐occurrence of natural‐adaptation and production‐trait targets within NWC‐dominated ROH islands is consistent with the multi‐wave admixture architecture described in Sections 2.2–2.5, wherein successive introgression pulses delivered both adaptive and production‐related variation on shared ancestral haplotypes.

#### 2.6.4 Multi‐Layer Candidate Integration

Integrating evidence across three layers—Layer 1 (any single‐statistic *FDR *< 0.05), Layer 2 (pathway support), and Layer 3 (ROH selection signal; Section 2.6.3)—we identified 223 strong candidate windows (218 unique genomic positions) across all three ancestry contrasts (Tables S17, S18; Figure S13C). The TIB versus NWC contrast contributed the largest share (169 windows), followed by TIB versus SYA (37) and TIB versus SHF (17), consistent with the longer tract‐length‐based age of NWC ancestry (Table 1).

At the pathway level, matched‐region permutation applied to the Layer 1 candidate set identified GPCR signaling (GO:0007186) as the only term surviving correction (permutation *p* = 1 × 10^−^^4^; FDR = 0.030; fold‐change = 2.19×; Figure S13A; Table S15). A complementary threshold‐free GSEA revealed a broader landscape: 50 pathways at FDR < 0.05 across 512 tested, led by Focal adhesion (*NES* = 1.57), Regulation of actin cytoskeleton (*NES* = 1.51), and Cell–cell adhesion (NES = 1.69), with 21 pathways significant in two or more contrasts (Figure S13B; Table S16).

##### 2.6.4.1 NWC‐Route Candidates (169 Windows, 31 Genes)

The NWC contrast contributed the largest share of candidates (Figure 6C; Table S18), consistent with its older introgression age (Table 1). A prominent cluster spans chr1: 74.2–75.6 Mb, encompassing VWF (12 windows; platelet adhesion and coagulation), TSPAN9 (12 windows; vascular cell‐surface organization), TEAD4 (8 windows; Hippo‐pathway transcription factor), FOXM1 (3 windows; cell‐cycle progression), ANO2 (6 windows; calcium‐activated chloride channel), and NTF3 (5 windows; neurotrophin‐3), overlapping with the hitchhiking cluster detailed in Section 2.6.3. Five NWC candidates carry Layer 2 GPCR pathway support: TSHR (5 windows), PRLHR, EZH1, RAMP2, and STK32B, accounting for the majority of the global GPCR enrichment signal. Additional candidates include INHBA (chr2), PCDH7 (chr4), and NELL1/SLC6A5 (chr5).

##### 2.6.4.2 SYA‐Route Candidates (37 Windows, 4 Genes)

The SYA contrast yielded the second‐largest set (Figure 6D). The dominant signal is CACNA2D1 (calcium voltage‐gated channel auxiliary subunit; 9 windows, chr1), with annotated roles in cardiac and neuronal function. AMY2A (pancreatic amylase; 3 windows, chr8) is a candidate for metabolic adaptation. HGF (hepatocyte growth factor; 1 window) and TSHR (1 window, cross‐route support) complete the set. These candidates are consistent with the organ weight/growth QTL enrichment identified for the SYA route in Section 2.6.2.

##### 2.6.4.3 SHF‐Route Candidates (17 Windows, 5 Genes)

The SHF contrast yielded the fewest candidates (Figure 6E), consistent with the recent and low‐intensity diffusion of SHF ancestry (∼15.3%). The most notable candidate is AGTR1 (angiotensin II type 1 receptor; 2 windows), which carries Layer 2 GPCR support and has annotated roles in blood pressure regulation and pulmonary vascular remodeling. TSHR (1 window) and CACNA2D1 (1 window) provide cross‐route support.

##### 2.6.4.4 Cross‐Route Convergence

TSHR is the only gene identified across all three ancestry contrasts [65] (7 windows total). CACNA2D1 appeared in both SYA and SHF contrasts (10 windows total). This pattern is consistent with convergent selection pressure on these loci from independent lineages, although residual demographic confounding not fully captured by the neutral null cannot be excluded (see also the enrichment‐level caveats in Section 2.6.2).

#### 2.6.5 Within‐Population nSL Signals

The between‐population composite detects completed or near‐completed sweeps producing population differentiation, but is insensitive to ongoing selection that extends haplotype homozygosity within TIB without altering allele frequencies relative to source populations. The within‐population nSL statistic [66] identified 709 top 1% windows with near‐zero overlap (2–3 windows) with the 223 calibrated candidates, confirming that it captures a distinct evolutionary process (Figure 6, nSL axis). Notable nSL peaks include *KCNA1/KCNA5/KCNA6* (hypoxic pulmonary vasoconstriction; chr1), *NDUFA9* (mitochondrial complex I), *HIF1AN* (HIF‐1α inhibitor; chr6), *ESRRG* (mitochondrial biogenesis; chr3), and the *HBB* cluster. These signals are consistent with soft sweeps on pre‐existing variation carried by multiple admixed ancestral sources. Because nSL has not been calibrated against the demographic null, these candidates remain hypothesis‐generating.

## 3 Discussion

Our analyses establish that Tibetan chickens derive from three irreducible ancestry sources—NWC, SYA, and SHF—whose contributions are organized along three complementary dimensions: ancestry identity, temporal layering, and functional partitioning. Resolving this architecture required two methodologically distinct approaches [48, 55, 67]: frequency‐based methods (qpWave/qpGraph) identified the source topology and confirmed the irreducibility of the three ancestry streams, whereas haplotype‐based methods (LAI/tract‐length dating) resolved the chronological ordering of admixture events and enabled route‐stratified selection analysis.

### 3.1 Temporally Layered Admixture and Its Historical Context

The drift‐based and haplotype‐based ancestry estimators act as complementary temporal lenses [68]: qpAdm recovers a larger share of the deep NWC and SHF layers eroded by recombination, while LAI accentuates recent gene flow, assigning SYA the largest share (54.7%) relative to NWC (28.1%) and SHF (17.1%). Because LAI relies on haplotype‐pattern matching, it is inherently most sensitive to long, recently introduced tracts; ancient tracts fragmented below detection resolution are systematically underassigned. This means LAI overstates the youngest layer (SYA) and understates the two older layers (NWC, SHF)—precisely the opposite of the bias that would falsely inflate the temporal depth of the model. The LAI‐based proportions, therefore, represent conservative lower bounds [57, 59] for the older ancestral contributions [57].

The four resolvable time‐scales (Table 1; Figure 4C,D) invite cautious historical interpretation. The deepest layer—NWC, constrained at >928 generations (>1392 years BP)—is broadly compatible with the inferred timescale of chicken dispersal into Inner Asia: paleoproteomics‐confirmed chicken eggshells along the Silk Road date to the fourth century BCE onward [4], while genomic analyses trace domestic chicken origins to G. g. spadiceus in southwestern China and Myanmar with subsequent translocation through South and Central Asia [69, 70]. Although no directly dated chicken remains have been reported from the Tibetan Plateau itself, these converging lines of evidence place the NWC founding layer within a plausible timeframe for early poultry husbandry reaching the plateau periphery [20, 58]. The major SYA influx at ∼514 generations (∼770 years BP; 95% CI: 740–811; robust across recombination rates of 1.2–3.11 cM/Mb, Figure S9A,B) coincides temporally with intensified trade along the Tea Horse Road during the Song through early Ming dynasties, though this association is correlational; we cannot exclude alternative scenarios such as multiple overlapping trade phases or continuous gene flow predating the major pulse. The intermediate exchange epoch (∼90–260 generations) is broadly contemporaneous with late Ming to Qing trans‐Himalayan trade expansion. We emphasize that all temporal associations are correlational, with confidence intervals spanning decades to centuries; dates represent approximate centers of mass rather than precise historical events.

The three ancestries exhibit distinct temporal signatures that are independently corroborated by tract fragmentation, ROH composition, and geographic decay patterns. SYA—the youngest and largest layer—retains the longest tracts (mean 0.65 Mb) and its ancestry proportion increases monotonically from short to long ROH (57% → 69%), because long ROH segments, being least fragmented by recombination, preferentially retain the most recently introgressed haplotypes. Geographically, SYA proportions decay steeply from the Sichuan–Yunnan source center (*ρ* = −0.73), and SYA‐ROH frequency mirrors this spatial gradient (*ρ* = −0.82; Figure 4G). By contrast, the SHF ancestry presents the oldest detected ancient component (928 generations) yet the most fragmented tracts (mean 0.24 Mb), and its contribution remains low and stable across ROH length classes—consistent with sustained low‐intensity diffusion along the southern Himalayan foothills over a prolonged period, though a single ancient pulse followed by extended recombination cannot be excluded. SHF proportions show the steepest geographic decay (*ρ* = −0.87), indicating geographically localized penetration. NWC—as the resident founding layer—decreases reciprocally in ROH from short to long segments (∼36% → 22%), confirming progressive fragmentation by subsequent waves of external gene flow. Its true founding depth cannot be resolved by tract‐length methods, which measure when subsequent introgression fragmented resident haplotypes rather than when NWC ancestry was first established; the SHF ancient component (∼928 gen) provides a lower bound, while fastsimcoal2 estimates a deeper founding (∼3312 gen) subject to upward SFS‐based bias (Methods 4.7.5). Geographically, NWC shows the complementary pattern to both external sources, enriched in subpopulations farthest from either introgression center (*ρ* = +0.65; Figure 4F), and NWC‐ROH frequency increases with distance from the SYA source (*ρ* = −0.87; Figure 4H), confirming that the spatial and temporal gradients are two facets of the same admixture architecture [71].

This three‐layer model also helps explain conflicting prior classifications of Tibetan chickens: methods sensitive to recent gene flow emphasize SYA, while those capturing deeper shared drift recover NWC and SHF. Some contradictions may also reflect genuine biological heterogeneity rather than analytical artifacts alone [48].

### 3.2 Geographic Dispersal Dynamics

The mirror‐image spatial structure described above (Section 3.1)—SYA and SHF declining, NWC increasing toward the plateau interior—sets the stage for examining how the two external routes differ in their fine‐scale dispersal dynamics. The SYA corridor shows discrete F‐branch decay steps from the lowland source (XSBN) toward the plateau interior, with elevated IBD at terminal populations (ZGC/MKC; Figure 5A,C), consistent with episodic, node‐structured gene flow possibly facilitated by valley‐linked trade networks. The SHF route exhibits smoothly declining IBD from the Himalayan foothills inward (Figure 5E–G), consistent with continuous stepping‐stone diffusion channeled through the Yarlung Tsangpo corridor [72, 73]. Two caveats apply: sparse sampling along the NWC corridor precludes a parallel route‐level analysis, and the EEMS‐supported corridor patterns cannot be fully disentangled from isolation‐by‐distance [53, 54, 72] (Section 2.3).

### 3.3 Selection Signals, Functional Partitioning, and Alternative Explanations

The twofold enrichment of calibrated composite signals above the neutral 1% tail across all three ancestry contrasts is consistent with a subset of differentiation signals exceeding demographic expectation [60, 74, 75, 76]. The ancestry‐stratified enrichment patterns—NWC enriched for hematology‐related genes, SYA for organ weight/growth, and SHF for cardiac muscle contraction—are hypothesis‐generating rather than definitive evidence of adaptive functional partitioning. The joint complementarity test (permutation *p* = 5 × 10^−^^4^; Figure 6B) provides statistical evidence that this three‐way partitioning cannot arise from stochastic variation or gene‐density bias alone, but these enrichments should be interpreted as statistical associations rather than exclusive biological systems; pleiotropy and pathway cross‐talk remain uncharacterized [77, 78]. The 223 strong candidates represent a prioritized set of hypotheses for functional investigation. The underrepresentation of SHF ancestry in ROH islands likely reflects its extreme tract fragmentation, which prevents aggregation into shared islands.

The route‐specific candidates align with prior high‐altitude findings in Tibetan chickens: the NWC‐enriched *VWF* was independently identified as a selective sweep target involved in platelet coagulation under hypoxia [28]; the SYA/SHF‐convergent *CACNA2D1* was previously reported as a calcium‐signaling adaptation candidate linked to HIF‐α–mediated hypoxia response [26]; and the SHF‐specific *AGTR1* has been associated with high‐altitude pulmonary edema susceptibility [79]. Within‐population nSL peaks at *HIF1AN*, *KCNA5*, *INHBA*, and the *HBB* cluster, similarly implicate well‐characterized hypoxia‐response pathways [27, 80, 81, 82]. These convergences do not confirm causality but provide biological priors supporting the observed enrichment patterns.

Several alternative explanations merit consideration, organized by the dimension they challenge. Temporal model: continuous gene flow rather than discrete pulses could produce similar tract‐length distributions; however, fastsimcoal2 model comparison decisively favored the Pulse model [60] (Δ*AIC* = 7212), and the four‐component mixture model's superior BIC provides additional support for temporally distinct events. Source model: unsampled ghost lineages could contribute misattributed ancestry; however, qpWave rank tests consistently accepted rank = 2, and no candidate fourth source increased the rank. Spatial model: geographic gradients could reflect isolation‐by‐distance rather than corridor‐mediated dispersal; both processes likely contribute, and we cannot fully disentangle them with the current sampling design (Section 2.3). Functional partitioning: route‐specific enrichment patterns could arise from genomic architecture differences rather than ancestry‐specific selection; the matched‐region permutation and joint complementarity tests argue against this, but uncontrolled confounders cannot be excluded [83, 84, 85, 86].

### 3.4 Broader Implications

The multi‐layer validation framework—combining demographic null calibration, matched‐region permutation, threshold‐free GSEA, and ROH‐based cross‐validation—may be applicable to other admixed livestock species such as Tibetan pigs, yaks, and Central Asian sheep [5, 23, 87, 88]. For Tibetan chicken conservation and breeding, the ancestry‐stratified candidate gene list (Table S18), particularly the chr1: 74–75.7 Mb region harboring both altitude‐adaptation and production‐trait genes, may warrant targeted investigation. Direct extrapolation to wild high‐altitude specialists requires caution given the fundamentally different selection regimes operating in domesticated populations [1, 15].

### 3.5 Limitations

Beyond the caveats noted in preceding sections, three structural limitations qualify our inferences. First, LAI accuracy (∼89%–93%) showed a consistent directional bias (SYA→NWC misassignment: 15.2%); although this bias direction does not undermine the temporal model, ancestry proportions should be treated as model‐dependent estimates. Second, the composite selection score is most sensitive to strong, recent sweeps; polygenic adaptation and soft sweeps on standing variation would largely escape detection [83, 89, 90], and the 223 candidates therefore represent a lower bound. Third, the absence of ancient DNA from the Tibetan Plateau prevents direct calibration of admixture times against archaeological specimens; future archaeogenomic integration would substantially strengthen the temporal inferences [91].

### 3.6 Conclusions

Tibetan chicken adaptation to the plateau was not shaped by a single evolutionary trajectory but by the temporally structured convergence of three ancestries, each entering via geographically distinct routes and at resolvable time‐scales: NWC as a deep founding layer (>928 generations), SYA as the major recent influx (∼514 generations, correlating with Tea Horse Road trade), and SHF as sustained low‐intensity diffusion from the southern Himalayan foothills. These ancestry layers carry statistically distinguishable functional candidate sets—vascular/coagulation genes via NWC, calcium signaling and metabolic genes via SYA, and cardiac/pulmonary candidates via SHF—suggesting that high‐altitude adaptation in this species is a composite, human‐facilitated assembly of complementary genetic modules rather than a monolithic selective response. The analytical framework presented here—coupling formal source identification with demographically calibrated, ancestry‐stratified selection scans—provides a replicable approach for dissecting multi‐source adaptation in other admixed livestock populations [1, 87].

## 4 Materials and Methods

### 4.1 Sample Collection and Whole‐Genome Resequencing

Whole‐genome resequencing data from 1054 chickens were analyzed, comprising 535 samples newly sequenced in this study on the DNBSEQ‐T7 platform (MGI Tech, Shenzhen, China) with paired‐end 150 bp reads, from diverse indigenous Chinese breeds and 519 publicly available genomes (Table S1). All data met a minimum of 10× median sequencing depth. Clean reads were mapped to the chicken reference genome GRCg6a (GCF_000002315.6) using BWA‐MEM (v0.7.17). Variants were called using the GATK (v4.2) [92, 93] pipeline and subjected to stringent quality control using vcftools (v0.1.16) [94, 95] and PLINK (v1.9), filtering for biallelic autosomal single‐nucleotide polymorphisms (SNPs) based on quality, depth, call rate (>90%), and minor allele frequency (>0.05). Sample sizes ranged from 9 (LZ) to 56 (CTC, lzhc) individuals per Tibetan subpopulation; a summary of population‐level sample sizes and their inclusion in each downstream analysis is provided in Table S1B. The experimental process complied with the rules of the Animal Welfare Committee of the State Key Laboratory of Agricultural Biotechnology at China Agricultural University (License No.: XK257).

### 4.2 Analytical Framework Overview

Our analyses were organized by three objectives. First, to characterize population structure and identify candidate ancestry sources, we performed PCA, ADMIXTURE, ML phylogeny, and F‐branch analysis on all 1054 individuals (Section 4.3). Second, (a) to validate the admixture model and resolve its geographic structure, we applied f‐statistics, qpWave rank tests, qpAdm, qpGraph, topology weighting (Twisst), graph fitting (Treemix), EEMS, and D‐statistics (Sections 4.4–4.6); and (b) to resolve the temporal stages of admixture, we used local ancestry inference and tract‐length dating (Section 4.7). Third, to identify genomic regions potentially shaped by selection, we combined route‐stratified differentiation scans with neutral demographic calibration, permutation‐based enrichment testing, and ROH demographic control (Sections 4.8–4.10). Each section below describes the method, justifies key parameter choices, and notes limitations.

Methods operating on unphased genotypes (PCA, ADMIXTURE, f‐statistics) are inherently robust to unequal group sizes; groups with *n* < 5 were flagged in Table S1B. All block‐jackknife standard errors for f‐statistics, qpAdm, and qpWave used 5 Mb blocks (ADMIXTOOLS2 default), appropriate for chicken LD decay distances.

### 4.3 Population Structure and Gene‐Flow Analysis

#### 4.3.1 PCA, ADMIXTURE, and ML Phylogeny

The dataset was LD‐pruned using PLINK [96] (–indep‐pairwise 50 10 0.2; standard thresholds for population structure analysis); the pruned set was used for PCA and ADMIXTURE [47], whereas the unpruned set was retained for phylogeny and introgression tests. Population structure was investigated using PCA and unsupervised clustering with ADMIXTURE (v1.3.0; *K *= 2–12). Although CV error reached its minimum at *K* = 15 (Figure S2A), *K* = 4 was selected for display because it most clearly resolves the SYA–SHF split; higher *K*‐values further subdivide reference populations without altering the two‐lineage pattern within TIB (Figure S2B).

An ML phylogenetic tree was constructed using RAxML [97] (v8.2.12) under the GTRGAMMA model [97] on the full SNP alignment (987 taxa, 1,134,033 unique site patterns), rooted with Red Junglefowl (T). Bootstrap support was assessed with 100 rapid bootstrap replicates on a 10% site subsample (GTRCAT model; mapped onto the full‐data tree via RAxML‐NG v1.2.2). For the population‐level trees (Figure 1C and Figure 5B,E), Felsenstein bootstrap proportions were computed by pruning each bootstrap tree to one representative per population and counting the proportion supporting each bipartition.

#### 4.3.2 D‐Statistics, f4‐Ratio, and F‐Branch

Patterson's D‐statistics and f4‐ratio statistics were computed using Dsuite [44] (v0.5). For the within‐Tibet F‐branch analysis (Figure 1D), Dsuite Dtrios was run on all 11 Tibetan populations plus Red Junglefowl (T) as outgroup, with block‐jackknife partitioning (*k* = 20 genomic blocks). F‐branch values were derived using Dsuite Fbranch (*p*‐value threshold = 0.01) [44]. Statistical support was assessed via the *Zb* matrix; cells with *Zb *> 3 (∼*p* < 0.003) are considered significant.

### 4.4 Three‐Source Admixture Model Validation

#### 4.4.1 Data Partitions and Population Sets

All admixture‐model analyses used the admixtools [48, 49] R package (v2.0) with precomputed block‐jackknife f2 statistics (afprod = TRUE). We analyzed three matched panels: a population‐group panel, a TIB subpopulation panel (10 subpopulations), and a breed‐level panel (maxmiss = 0, blgsize = 0.01). Three wild Gallus species—Green Junglefowl (G. varius, *n* = 3), Grey Junglefowl (G. sonneratii, *n* = 3), and Ceylon Junglefowl (G. lafayettii, *n* = 3)—were newly incorporated as core safe outgroups; T, MRJ, and VN were retained as legacy outgroups and evaluated for treeness violations [98] (Figure S3B–D). White Leghorn was excluded based on prior f4 evidence of introgression into TIB.

#### 4.4.2 f‐Statistics, qpAdm, and Geographic Analysis

Admixture f3 statistics were computed for the pooled TIB and each subpopulation. qpAdm compared one‐ through four‐source models across six outgroup configurations; models were accepted when rank‐drop *p* ≥ 0.05 and all mixture weights ≥ −0.01. Geographic ancestry gradients were quantified using f4(W, subpopulation; source_A, source_B) averaged across the three safe outgroups and correlated with longitude (Pearson's r).

#### 4.4.3 qpWave Rank Tests

qpWave assessed source independence using a filtered NWC proxy (NWC_pure = TP + BYC + JYC, excluding heterogeneous HTC). We tested {SYA, SHF, NWC_pure} and {SYA, SHF, NWC_pure, TIB} across eight nested outgroup sets (3safe to 3safe+VN+T+MRJ). Legacy outgroups were added sequentially to increase the degrees of freedom of the f4 matrix beyond the single d.f. available with three safe outgroups alone. Rejection of rank = 1 would indicate three irreducible ancestry streams; acceptance of rank = 2 would confirm that TIB lies within the three‐source space. Potential fourth‐source signals were diagnosed via f4(GreenJF, Source; LegacyOG, TIB_subpop) cross‐interaction statistics.

#### 4.4.4 qpGraph Automated Topology Search

Pure‐source proxies were constructed by ranking individuals within each source by mean f4 values across safe outgroups and pooling the top 15 per group. find_graphs (ADMIXTOOLS2) was run on six‐population sets (GreenJF, CeylonJF, three proxies, one TIB target) with *k* = 0–3 admixture events (stop_gen = 500, stop_gen2 = 50, numgraphs = 10, seed = 42 + *k*). Models with worst |*Z*| < 3 were accepted.

We also tested within‐source splits (SYA: SJ vs. XS; SHF: BJST vs. INDIA; NWC: TP vs. BYC) under two outgroup sets. Any split that increased rank was followed up with f4 contrasts, four‐source qpAdm, and replacement tests.

### 4.5 Geographic Corridors and Barriers to Gene Flow

#### 4.5.1 Topology Weighting (Twisst)

Per‐window gene trees were inferred from majority‐allele consensus sequences using PhyML [99] (BIONJ, HKY85). Twisst [51] (v0.2) then quantified support for alternative quartet topologies (TIB, SYA, SHF, NWC) across 50‐SNP non‐overlapping windows. Control analyses using VN and T as outgroups confirmed rooting consistency. Topology weights were reported as genome‐wide means with 95% CIs from block‐bootstrap resampling.

#### 4.5.2 Graph Modeling (Treemix)

Treemix (v1.13) [52] inferred the ML population tree and migration edges (−*k* 1000). The optimal number of edges (*m* = 2 with *T*; *m* = 3 with VN) was selected from *m* = 0–5. Inferred edges were validated by targeted f4 contrasts and F‐branch clustering (Figure S5C–E).

#### 4.5.3 Estimated Effective Migration Surfaces (EEMS)

EEMS [53, 54] was applied to a genetic dissimilarity matrix (1 123 489 SNPs; 982 individuals, 81 locations) on a 362‐deme triangular grid. The MCMC ran for 8 × 10^6^ iterations (4 × 10^6^ burn‐in) with default priors; convergence was confirmed by acceptance‐rate diagnostics.

#### 4.5.4 Patterson's D‐Statistics for Corridor Validation

D‐statistics were computed for population triplets along candidate corridors to test specific barrier and connectivity hypotheses identified by EEMS. Three geographic features were tested: the Himalayan main ridge (GB–CTC–INDIA), the Yarlung Tsangpo Grand Canyon corridor (GB–LZ–LBC), and the Yunnan–Guizhou Plateau connectivity (LN–DL–DWS). Significance was assessed using block‐jackknife standard errors.

### 4.6 Local Ancestry Inference [68]

#### 4.6.1 LAI Pipeline

The VCF was phased using Beagle [100, 101] (v5.5) with a uniform‐rate genetic map (3 cM/Mb) [102]. LAI was performed on 309 TIB individuals using Flare [57] (v0.5.3) with the same genetic map, which accommodates multi‐way admixture via an HMM framework with per‐site posterior probabilities. Reference panels were constructed from individuals with the highest target‐ancestry coefficients (ADMIXTURE *K* = 3), yielding balanced panels (SYA: 79, SHF: 78, NWC: 80; Table S11) to minimize prior bias. Population‐level ancestry proportions were computed by averaging posterior probabilities across individuals and sites (posterior‐mean/soft‐call summary).

#### 4.6.2 LAI Validation and Sensitivity

Inference accuracy was assessed through three approaches. (i) Pseudo‐admixed benchmark: Four held‐out individuals per ancestry were used to construct in silico mosaic genomes at six prescribed ratios; per‐site accuracy reached ∼88%–90% in the real‐like scenario (69/11/20), with SYA→NWC as the dominant misassignment (15.2%; Figure S7A,B). (ii) Leave‐one‐out cross‐validation: each breed was removed from the reference panel and re‐classified, confirming high accuracy (SYA 92.9%, SHF 88.7%, NWC 89.2%; Figure S7C,D). (iii) Reference‐panel downsampling: ancestry estimates remained stable at ≥50% panel retention (Pearson *r* > 0.99; Figure S7E), with degradation only at 25%.

Genome‐wide assignment confidence (maximum posterior probability per site) exceeded 0.9 at 49.4% of sites and 0.7 at >92% (Figure S11A,B). Pairwise F_ST between panels (0.0326–0.0388) predicted the observed SYA→NWC misassignment bias. Only sites with maximum posterior (hard‐call) assignment probability >0.7 were retained for downstream ancestry‐specific analyses.

#### 4.6.3 Chromosome Painting and Geographic Correlation

Ancestral mosaics were visualized using RIdeogram [103] (v0.2.2; Figure 4A,B). Great‐circle distances between each TIB subpopulation and each source center (SYA: 26.86°N, 100.23°E; SHF: 28.63°N, 82.44°E; NWC: 38.12°N, 96.79°E) were computed, and ancestry proportions (*n* = 9 subpopulation medians) were regressed against distance (LOESS with 95% bands; Spearman's *ρ*).

### 4.7 Admixture Dating

#### 4.7.1 DATES Admixture‐LD Analysis

DATES [58, 59] (v753) was applied to all 309 TIB individuals for three pairwise source combinations (NWC–SYA, NWC–SHF, SYA–SHF) using the same uniform‐rate genetic map (Section 4.6.1) and fine‐resolution parameters (binsize = 0.0002 M). A two‐exponential decay model was fitted to the LD covariance curves (Figure S8A). Per‐individual estimates were filtered for degenerate fits (recent component ≤1 generation) and outliers (>3 × IQR), retaining *n* = 284–292 per pair. For the cross‐method comparison (Figure 4C), each pair's ancient‐component median and bootstrap 95% CI (10 000 resamples) were reported.

#### 4.7.2 Tract‐Length Mixture Model Specification

Ancestry tract lengths from Flare were extracted for TIB individuals on macrochromosomes (chr 1–5), which have relatively uniform recombination rates. For each ancestry, k‐component truncated exponential mixture models (*k* = 1–4) were fitted:

$$f\left(L\right)=\Sigma_{i}\pi_{i}\;\cdot \;\lambda \;exp\left(-\lambda_{i}L\right)/\left[exp\left(-\lambda_{i}L_{min}\right)\;-\;exp\left(-\lambda_{i}L_{max}\right)\right]$$

where *λ*_i_ = *t*_i_ × *r*, [*L*_min_, *L*_max_] = [0.005, 50] Mb, and a uniform recombination rate of 3 cM/Mb [102], a conservative value informed by the genome‐wide average of 3.11 cM/Mb and the macrochromosome median of 2.8 cM/Mb [43] was used. Parameters were estimated via EM with 200 random restarts. BIC overwhelmingly favored *k* = 4 (ΔB*IC*_3_→_4_ = 66–738; LRT *p* < 10^−^^1^ ^5^ for all ancestries; Figure S8C). Sensitivity analyses spanned recombination rates (1.2–3.11 cM/Mb; Figure S9A,B), minimum tract‐length cutoffs (0.005–0.02 Mb; Figure S9C), and generation times (1–2 years/gen; Figure S9D,E). 95% CIs were obtained by bootstrapping individuals (1000 replicates).

#### 4.7.3 Binary Reclassification for Resolving NWC Founding Depth

Because NWC tract‐length distributions reflect fragmentation by subsequent introgression rather than NWC entry time, we performed binary reclassification: NWC+SYA tracts were merged into “non‐SHF” super‐tracts and NWC+SHF into “non‐SYA” super‐tracts, each fragmented by only one external source. The same mixture model (Section 4.7.2) was fitted to each set (Figure S8D).

#### 4.7.4 Flare Global Admixture‐Time Parameter

Flare's per‐chromosome transition‐rate parameter T was computed across 13 macrochromosomes (chr 1–8, 10–14; two chromosomes excluded for EM convergence failure: *T* > 5000 gen). The median T with nonparametric bootstrap 95% CI (10 000 resamples) provides an independent check on the tract‐length and DATES estimates (Figure 4C).

#### 4.7.5 fastsimcoal2 [60] Demographic Model and Known Biases

The demographic model (Section 4.10.1) served two purposes: model selection between pulse and continuous‐migration histories, and parameterization of neutral simulations for selection scans (Section 4.10.2). SFS‐based timing estimates were systematically deeper than tract‐length dates, consistent with the known upward bias of SFS‐based coalescent methods in species with pervasive background selection; these estimates were treated as order‐of‐magnitude upper bounds (see Discussion 3.1).

#### 4.7.6 Ancestry‐Stratified ROH Analysis

ROH were detected using PLINK [71, 96] (–homozyg; standard WGS thresholds: ≥50 SNPs, ≥500 kb, density ≤ 1/50 kb, gap ≤ 1000 kb, window 50 SNPs, ≤1 het/window). ROH segments were classified as short (<1 Mb), mid (1–5 Mb), and long (>5 Mb), and each segment was assigned a dominant ancestry from LAI. Geographic correlations between ROH frequency and source‐center distance were tested by Spearman's *ρ*. Per‐individual inbreeding coefficients (*f*_ROH_ = total ROH/autosomal genome length) were computed along each dispersal corridor.

### 4.8 Fine‐Scale Diffusion Analysis

#### 4.8.1 Route‐Specific F‐Branch and Chromosome‐Jackknife

Route‐specific F‐branch analysis (Figure 5A) was performed by running Dsuite Dtrios on the SYA‐corridor population subset. Uncertainty was estimated via chromosome‐jackknife (*n* = 32 autosomes); error bars in Figure 5A represent ±1.96 SE. Values, Zb‐scores, and SEs are in Table S23.

#### 4.8.2 IBD Analysis

Pairwise IBD (PI_HAT) was estimated using PLINK (–genome) [96] on populations along each corridor. SYA route: XSBN, ZY, XS, SJ, NC, ZGC, MKC. SHF route: INDIA, CTC, ZHJ, LZ, GB. IBD matrices were visualized as heatmaps ordered by along‐corridor position (Figure 5C,F).

#### 4.8.3 Phylogenetic Sub‐Analysis

Route‐specific ML trees were constructed using RAxML (v8.2.12) under the ASC_GTRGAMMA model with Lewis‐style ascertainment‐bias correction (required because route subsets contain only polymorphic sites from the full alignment). Per‐route population composition is listed in Table S1B. Trees were rooted with T and VN as outgroups. Bootstrap support values in Figure 5B,E derive from the full‐dataset pruning approach (Section 4.3.1; 100 replicates).

#### 4.8.4 Route‐Anchored Regression

Route‐anchored regression quantified the decay of genetic affinity (mean PI_HAT) with corridor distance, using ZGC (SYA route) and GB (SHF route) as terminal references. Significance was assessed via Pearson's r (*n* − 2 d.f.; Figure 5D,H).

### 4.9 Selection Scan Workflow and Candidate‐Gene Annotation

Genetic divergence between TIB and each source population was quantified in 10 kb non‐overlapping windows using Pixy (v1.2.1) [104] for F_ST [105, 106] and π, and Selscan [66] (v2.0) for XP‐EHH (normalized per chromosome). For each contrast, we computed *z*‐scores of F_ST, log_2_(π_POP/π_TIB), and per‐window maximum normalized XP‐EHH. All thresholds were calibrated against the neutral demographic null (Section 4.10). Candidate genes were annotated using the GRCg6a GFF3 gene set (23 603 genes), with GO Biological Process and KEGG pathway mappings obtained via org.Gg.eg.db (Bioconductor) and QTL annotations from the Animal QTLdb (Release 58; GRCg6a coordinates). Carrying frequencies of candidate alleles were computed across TIB geographic subgroups with bootstrap CIs.

### 4.10 Selection Signal Validation

#### 4.10.1 Demographic Model Fitting

Two models were fitted to six pairwise folded 2D‐SFS (projected to 20 haploid chromosomes per population) using fastsimcoal2 (v2.8). The Pulse model (14 parameters) used the qpGraph‐verified topology (SYA, (SHF, NWC)); the Continuous Migration model (15 parameters) replaced the SHF pulse with bidirectional migration and TIB exponential growth. Each model was optimized 100 times; selection used AIC (generation time = 1.5 years). Model selection compared the two topologies using AIC. Model‐expected 2D‐SFS residuals were inspected to verify that the chosen null was conservative for positive selection detection (Figure S12A).

#### 4.10.2 Neutral Coalescent Simulation

Under the best‐fit Pulse model, msprime (v1.2) [61, 62] simulated 5 Mb segments (124 per replicate, proportionally allocated across chr 1–5 with chromosome‐specific recombination rates of 3–3.5 × 10^−^^8^ per bp per generation [102]; *µ* = 1.5 × 10^−^^9^ per bp per generation) [107]. Sample sizes matched empirical data. A total of 32 replicates yielded 1 837 000 null 10 kb windows for F_ST/*π*‐ratio and 1 469 600 for XP‐EHH (after 500 kb edge trimming). Null composite scores were computed as *z*(F_ST) + *z*(−*π*‐ratio) + *z*(XP‐EHH).

#### 4.10.3 *Z*‐Score Calibration and Parametric Bootstrap

Observed statistics were *z*‐score normalized; calibrated *p* = P(z_null ≥ z_obs). BH‐FDR correction [108] was applied per contrast (*q* < 0.05). Genomic inflation factors (λ_GC = 0.76–0.78) confirmed a conservative null (Figure S12B–D). Robustness was verified by parametric bootstrap (20 parameter sets, ±20% perturbation, 50 000 windows each; *z*‐score threshold *CV* < 1%).

#### 4.10.4 Matched‐Region Permutation Enrichment

Windows (96 095) were annotated for gene density and GC content (bedtools v2.30.0 [109]; GRCg6a). For each of 301 GO/KEGG pathways (≥5 candidate genes), 10 000 permutations replaced candidate windows with size‐matched random windows of similar gene density (±20%) and GC content (±5 pp). *BH‐FDR* was applied across all 301 pathways.

#### 4.10.5 GSEA Continuous Enrichment

 fgsea (v1.24.0) [110, 111] was run on genes ranked by maximum composite selection score (512 pathways, gene set size 15–500, 10 000 permutations; significance: *BH‐FDR* < 0.05).

#### 4.10.6 Ancestry‐Stratified Enrichment and Joint Complementarity Test

Top‐5% candidate windows (empirical composite threshold) were classified by dominant LAI ancestry. The top‐5% threshold was used instead of the stricter FDR criterion because the latter retains too few windows per route for pathway‐level testing. Fisher's exact test compared enrichment per route against the other two routes combined. The joint complementarity test permuted route labels 10 000 times on a 3 × 3 OR matrix (routes × functional categories), using the diagonal OR product as test statistic (observed = 29.19 vs. null 99th percentile = 7.07; *p* = 5 × 10^−^^4^).

#### 4.10.7 ROH Island Identification and Demographic Control

ROH islands were defined as genomic regions where per‐SNP ROH frequency exceeded the 95th percentile [71] across all TIB individuals (58 islands; 50 NWC‐ancestry, 8 SYA‐ancestry; 3967 windows). Each island was cross‐referenced with calibrated composite *p*‐values under the ancestry‐appropriate contrast. KS tests assessed *p*‐value deviation from uniformity; Fisher's exact tests compared FDR‐significant window fractions inside versus outside islands (Figure S14). Significant ROH islands were annotated with chicken QTL data (Animal QTL database, Release 58; 26 349 autosomal entries; ±100 kb flanks); QTL category enrichment was tested by Fisher's exact test with BH‐FDR. Because demographic drift and inbreeding could elevate ROH burden genome‐wide, only ROH islands that independently satisfied the calibrated neutral composite null (Layer 1; Section 4.10.3) were retained as adaptation candidates (13/58 islands; *OR* = 10.91, *p *= 3.4 × 10^−^^1^ ^6^); this convergent‐evidence criterion filters drift‐driven ROH without requiring an explicit ROH‐based null simulation.

#### 4.10.8 nSL Within‐Population Scan

nSL was computed for TIB using Selscan (v2.0) [66, 90], normalized per chromosome. Candidate windows were defined as the top 1% of |nSL| values. Overlap with calibrated Layer 1 candidates was quantified. Because nSL was not calibrated against the demographic null, these candidates remained hypothesis‐generating.

#### 4.10.9 Three‐Layer Integration

Strong candidates required convergent evidence: Layer 1 (any calibrated statistic at BH‐FDR *q* < 0.05), Layer 2 (gene in a significantly enriched pathway), and/or Layer 3 (ROH island with selection signal exceeding demographic expectation). Windows outside ROH islands required Layers 1 + 2; windows within ROH islands required Layers 1 + 3.

### 4.11 Statistical Analysis

#### 4.11.1 Data Pre‐Processing

Pre‐processing details for each analysis were described in the corresponding methods sections (Section 4.1 for QC and Section 4.3.1 for LD pruning; Sections 4.7.1–4.7.2 for tract‐length and admixture‐LD filtering; Section 4.10.1 for SFS projection). No mathematical transformations were applied to genotype data. Outlier samples were identified and removed based on PCA projection.

#### 4.11.2 Data Presentation

Point estimates were reported with associated uncertainty measured as follows: admixture proportions (qpAdm) as mean ± block‐jackknife SE; f‐statistics and D‐statistics as point estimate ± SE with |*Z*| > 3 as significance threshold; topology weights (Twisst) as genome‐wide mean with 95% CI from block‐bootstrap; admixture dates (DATES) as median with bootstrap 95% CI (10 000 resamples); tract‐length dating as component timing with 95% CI from 1000 individual‐level bootstraps; geographic correlations as Spearman's *ρ* with exact *p*‐value (*n* = 9); selection statistics as calibrated z‐scores and BH‐FDR *q*‐values; enrichment tests as OR with permutation *p‐*value and BH‐FDR *q*‐value; route‐anchored regressions as Pearson's r (*n* − 2 d.f.); ROH inbreeding as f_ROH per individual or subpopulation mean ± SD; phylogenetic support as Felsenstein bootstrap proportions (≥50 shown). Unless otherwise specified, significance was defined at *α* = 0.05 (two‐sided).

#### 4.11.3 Sample Sizes

The dataset comprised *n* = 1054 chickens (535 newly sequenced, 519 public). Population sizes ranged from *n* = 9 (LZ) to *n* = 56 (CTC, lzhc) for TIB subpopulations, and *n* = 81 (SHF), *n* = 86 (NWC), *n* = 134 (SYA) for source populations. LAI used 309 TIB individuals with balanced reference panels (SYA: 79, SHF: 78, NWC: 80). A complete per‐analysis sample‐size summary is provided in Table S1B.

Sample‐size robustness was confirmed through four approaches: (i) qpAdm three‐source models passed for all 10 TIB subpopulations individually (*n* = 9–56; all *p* > 0.05; Table S9); (ii) LAI downsampling preserved estimates at ≥50% panel retention (*r* > 0.99; Section 4.6.2); (iii) SFS projection to uniform *n* = 20 haploids eliminated size asymmetry for demographic modeling (Section 4.10.1); (iv) the NWC corridor was excluded from route analyses due to inadequate sampling (Section 4.8).

#### 4.11.4 Statistical Tests and Multiple Testing Correction

Detailed statistical procedures are described within each analytical section; key tests and corrections are summarized here.

Admixture model testing (Section 4.4). qpAdm rank‐drop chi‐square (acceptance: *p* ≥ 0.05, weights ≥ −0.01); qpWave sequential rank tests (rejection of rank = 1 at *p* < 0.05; acceptance of rank = 2 at *p* ≥ 0.05); qpGraph adequacy (worst |*Z*| < 3). One‐ through four‐source models compared across six outgroup configurations (Table S3).

Admixture dating (Section 4.7). BIC and LRT for mixture‐model component selection (*k* = 1–4; BIC favored *k* = 4; LRT *p* < 10^−^^1^ ^5^). fastsimcoal2 model comparison by AIC (Δ*AIC* = 7212).

Selection scans (Section 4.9–4.10). *Z*‐score calibration against demographically simulated null (Sections 4.10.2–4.10.3); BH‐FDR per contrast (*q* < 0.05). Matched‐region permutation (10 000 permutations) and GSEA (10 000 permutations) with BH‐FDR across all pathways. Joint complementarity permutation test (10 000 permutations; Section 4.10.6). ROH island enrichment by Fisher's exact test with BH‐FDR (Section 4.10.7).

Geographic and corridor analyses (Sections 4.5, 4.6.3, and 4.8). D‐statistics with block‐jackknife SE; Spearman's *ρ* (*n* = 9) with exact *p*‐values; Pearson's r (*n* − 2 d.f.) for route‐anchored regressions. EEMS convergence confirmed by acceptance‐rate diagnostics.

#### 4.11.5 Statistical Software

Software packages and libraries used across all analyses in this study are summarized in Table 2. Statistical panels in the main and supporting figures were generated in R or Python. Figure 1A was generated using Microreact [45] (https://microreact.org/); the Microreact basemap (Maps, Mapbox and OpenStreetMap) is attributed in the Figure 1 caption. Figure 5 map panels (A, B, and E) were produced in ArcGIS using its default elevation map as the basemap; the Esri license statement (“Map image is the intellectual property of Esri and is used herein under license. Copyright 2026 Esri and its licensors. All rights reserved.”) is included in the Figure 5 caption. Composite layout and annotation were performed using Microsoft PowerPoint (Microsoft 365) for Figures 1, 3, 4, and 5, and using the cowplot R package for Figure 2, Figure 6, and all supporting figures. The Table of Contents image was drawn in Adobe Illustrator (2024 release). No generative‐AI image tools were used to create figure images; except for the ArcGIS default elevation basemap disclosed for Figure 5, no third‐party graphical assets, reused/adapted published figures, logos, or trademark images were incorporated.

**TABLE 2:** Software, versions, and primary applications used across all analyses in this study.

Software	Version	Purpose
PLINK	v1.9	PCA, LD pruning, ROH detection, IBD estimation
ADMIXTURE	v1.3.0	Unsupervised ancestry clustering (K = 2–12)
admixtools (R)	v2.0	f‐statistics, qpAdm, qpWave, qpGraph
Dsuite	v0.5	D‐statistics, F‐branch analysis
RAxML/RAxML‐NG	v8.2.12 / v1.2.2	ML phylogeny and bootstrap
Treemix	v1.13	ML population graph with migration edges
PhyML	v3.3	Per‐window gene trees for Twisst (BIONJ, HKY85)
Twisst	v0.2	Topology weighting
EEMS	—	Effective migration surface estimation
Beagle	v5.5	Haplotype phasing
Flare	v0.5.3	Local ancestry inference
DATES	v753	Admixture‐LD dating
fastsimcoal2	v2.8	Demographic model fitting (SFS‐based)
msprime	v1.2	Neutral coalescent simulation
Pixy	v1.2.1	Windowed F_ST and π estimation
Selscan	v2.0	XP‐EHH and nSL computation
bedtools	v2.30.0	Window annotation (gene density, GC content)
fgsea (R)	v1.24.0	Gene set enrichment analysis
RIdeogram (R)	v0.2.2	Chromosome painting visualization
rEEMSplots (R)	—	EEMS visualization
R	v4.4.3	Statistical computing environment
Python	v3.9+	Statistical analysis scripts and Python‐generated figure panels
BWA‐MEM	v0.7.17	Read alignment to GRCg6a
GATK	v4.2	Variant calling
vcftools	v0.1.16	Variant filtering
ggplot2 (R)	v3.4.4	Statistical figure panels generated in R
Adobe illustrator	2024 release	Table of contents image
PowerPoint	Microsoft 365	Composite layout and annotation (Figure 1, Figure 3, Figure 4, and Figure 5)
cowplot (R package)	1.2.0	Composite layout and annotation (Figure 2, Figure 6, and all supporting figures)
Microreact	https://microreact.org/	Figure 1A sampling map
ArcGIS	Default elevation map	Figure 5 map panels

During the preparation of this manuscript, the authors used Claude Code (Anthropic, claude‐opus‐4.5) to assist with code development for statistical analyses and figure generation, as well as language editing of the manuscript text. All AI‐assisted outputs were reviewed, verified against primary data, and edited by the authors. The authors take full responsibility for the scientific content, interpretation, and conclusions of the publication. No generative AI tools were used to generate primary data, design experiments, or draw scientific conclusions.

## Author Contributions

Conceptualization, Hao Zhang; Methodology, Li Zhu, and Bo Zhang; Software, Li Zhu; Validation, Tenzin Ngodroup, Li Zhu, and Bo Zhang; Formal Analysis, Zongyi Zhao; Investigation, Ruixue Nie, Hailu Fan, and Tenzin Ngodroup; Resources, Ruixue Nie, Hailu Fan, Tenzin Ngodroup, and Hongbin Pan; Data Curation, Ruixue Nie; Visualization, Zongyi Zhao and Bo Zhang; Writing – Original Draft, Zongyi Zhao; Writing – Review & Editing, Zongyi Zhao, Tenzin Ngodroup, Li Zhu, Bo Zhang, and Hao Zhang; Supervision, Hao Zhang; Project Administration, Hao Zhang; Funding Acquisition, Hongbin Pan, Bo Zhang, and Hao Zhang.

## Funding

This study was supported by the National Key Research and Development Program of China (grant number 2023YFF1002600), the Chinese Universities Scientific Fund (2025TC067, 2025TC135), and the 2115 Talent Development Program of China Agricultural University.

## Ethics Statement

All animal procedures in this study were reviewed and approved by the Animal Welfare Committee of the State Key Laboratory of Agricultural Biotechnology, China Agricultural University (Beijing, China), under Approval No. XK257. Blood and tissue sampling of Tibetan chickens and reference populations was conducted in accordance with the institutional guidelines for the care and use of laboratory animals and with the relevant national regulations of the People's Republic of China (Laboratory Animal—Guideline for Ethical Review of Animal Welfare, GB/T 35892‐2018). All efforts were made to minimize animal discomfort during sample collection. No animals were sacrificed specifically for this study; publicly available genome data used as reference populations are listed in Table S1.

## Conflicts of Interest

The authors declare no conflicts of interest.

## Supporting information




**Supporting File 1**: advs75536‐sup‐0001‐SuppMat.docx.

**Supporting File 2**: advs75536‐sup‐0002‐Tables.zip.

## Data Availability

All data generated in this study have been deposited in the National Genomics Data Center (NGDC) under the BioProject accession PRJCA049597 and are publicly accessible at https://ngdc.cncb.ac.cn/bioproject/browse/PRJCA049597. Publicly available reference genomes used in this study are listed in Table S1.
