# Supplementary material for: Large‐Scale Genomics Reveals Three‐Source Ancestry and Layered Adaptation to High Altitude in Tibetan Chickens
Source: Adv Sci (Weinh). 2026 May 12;13(42):e22994. doi: 10.1002/advs.202522994 (PMC13335608; doi:10.1002/advs.202522994)
Supplement: Supplementary file 1 — Supporting File 1: advs75536‐sup‐0001‐SuppMat.docx. [file ADVS-13-e22994-s002.docx]

Supporting Information

Large-Scale Genomics Reveals Three-Source Ancestry and Layered Adaptation to High Altitude in Tibetan Chickens

Zongyi Zhao, Ruixue Nie, Tenzin Ngodroup, Li Zhu, Bo Zhang, Hongbin Pan, and Hao Zhang*

**Supplementary Figures**

**Figure S1**


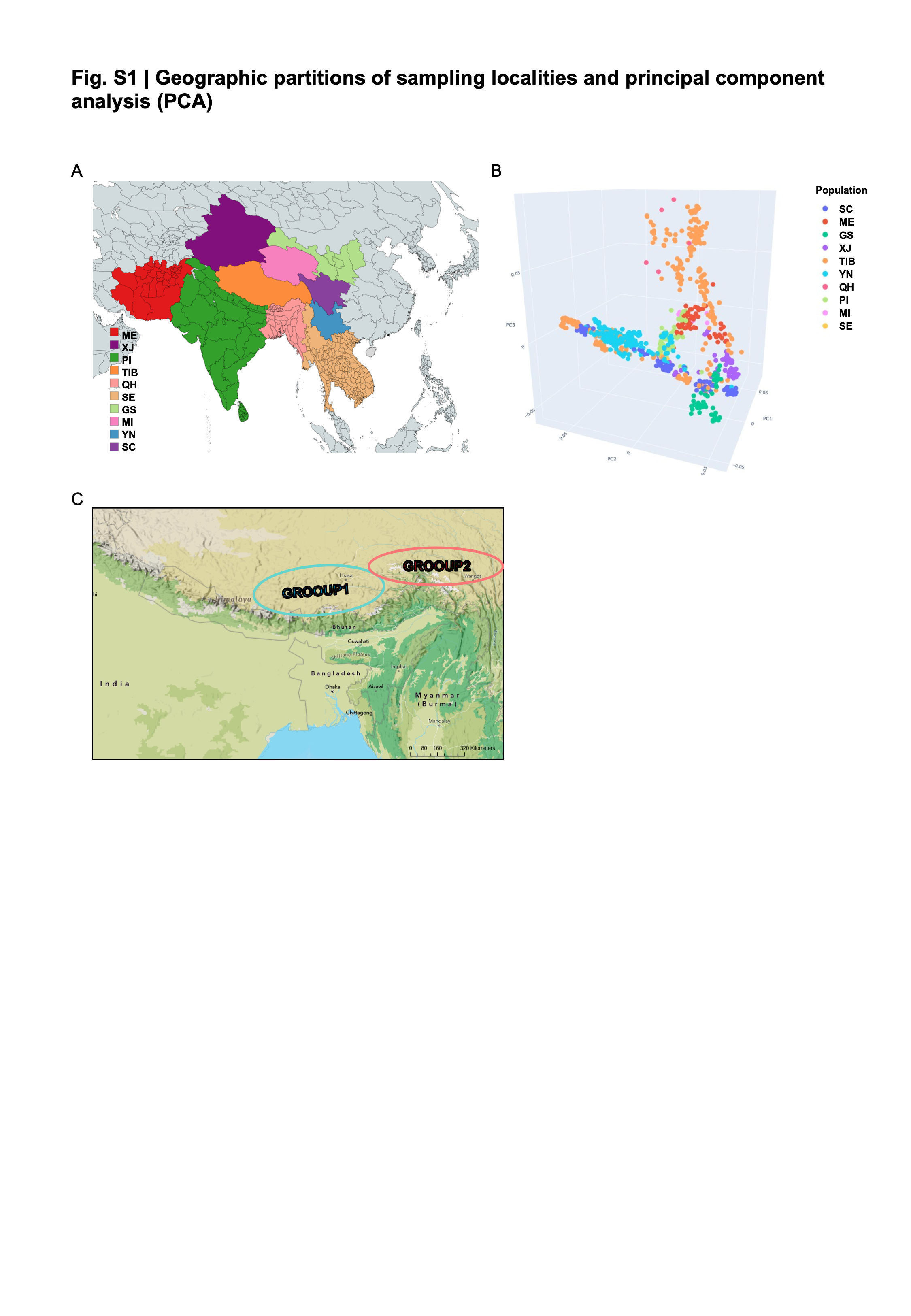


**Figure S1. Geographic blocks and global PCA of all study populations.** (A) Map showing the assignment of 55 reference populations to nine geographic blocks; basemap: © OpenStreetMap contributors (https://www.openstreetmap.org/copyright). (B) Three-dimensional global PCA with all 1,054 individuals; PC1, PC2, and PC3 explain 24.35%, 21.83%, and 14.16% of the total variance, respectively. (C) PCA zoomed to the Tibet block and immediate neighbors; basemap: ArcGIS default elevation map. Map image is the intellectual property of Esri and is used herein under license. Copyright © 2026 Esri and its licensors. All rights reserved.

Statistical panels were generated in R or Python; composite layout and annotation were performed using the cowplot R package.

**Figure S2**


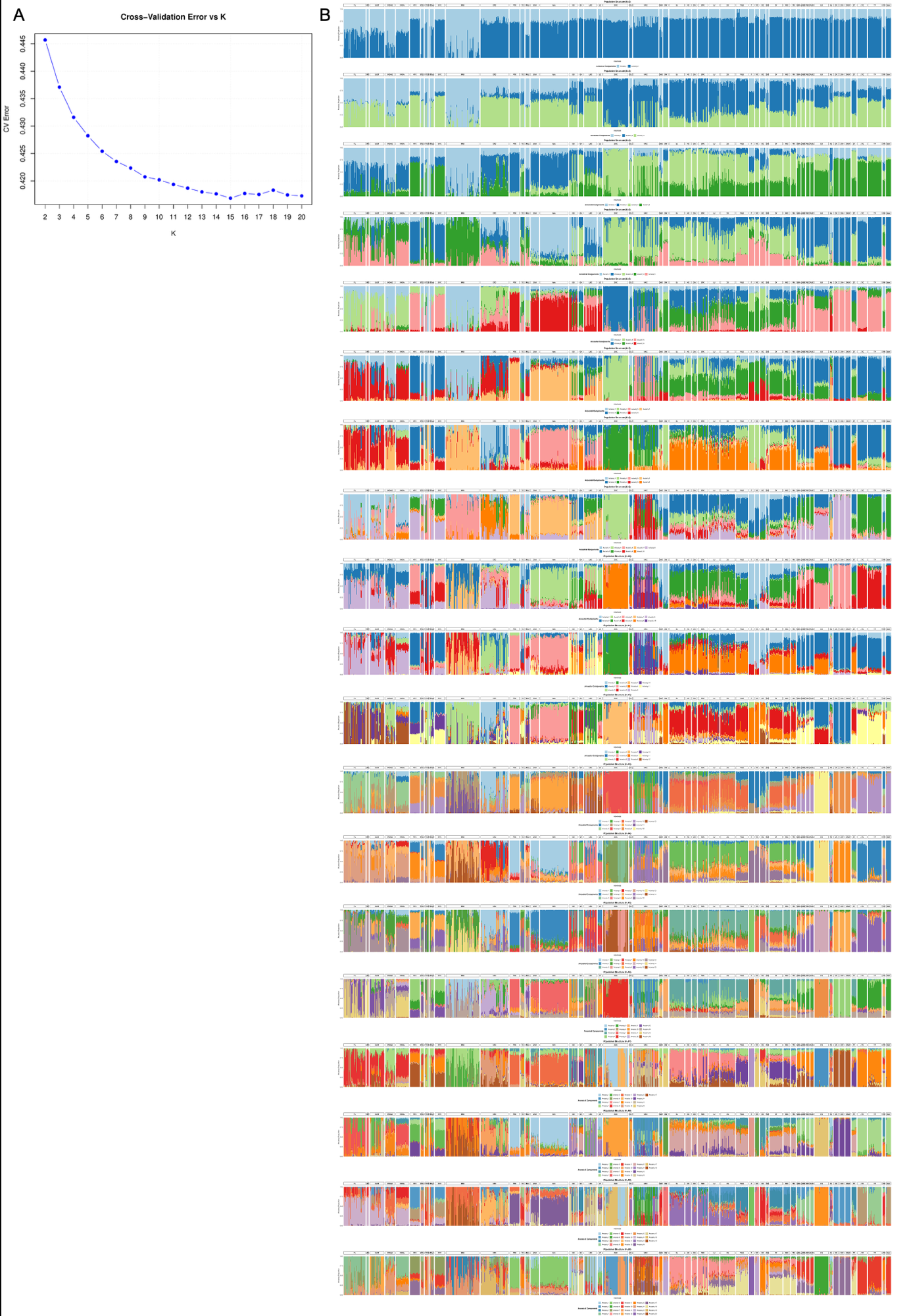


**Figure S2. ADMIXTURE cross-validation error across K = 2–20.** CV error decreases monotonically and reaches its minimum at K = 15. K = 4 is selected for display in Figure 1E because it clearly resolves the SYA–SHF split and the NWC component relevant to the three-source admixture model; higher K values subdivide reference populations but do not alter the two-lineage pattern within TIB. (A) CV error curve. (B) ADMIXTURE bar plot at selected K values.

Statistical panels were generated in R or Python; composite layout and annotation were performed using the cowplot R package.

**Figure S3**


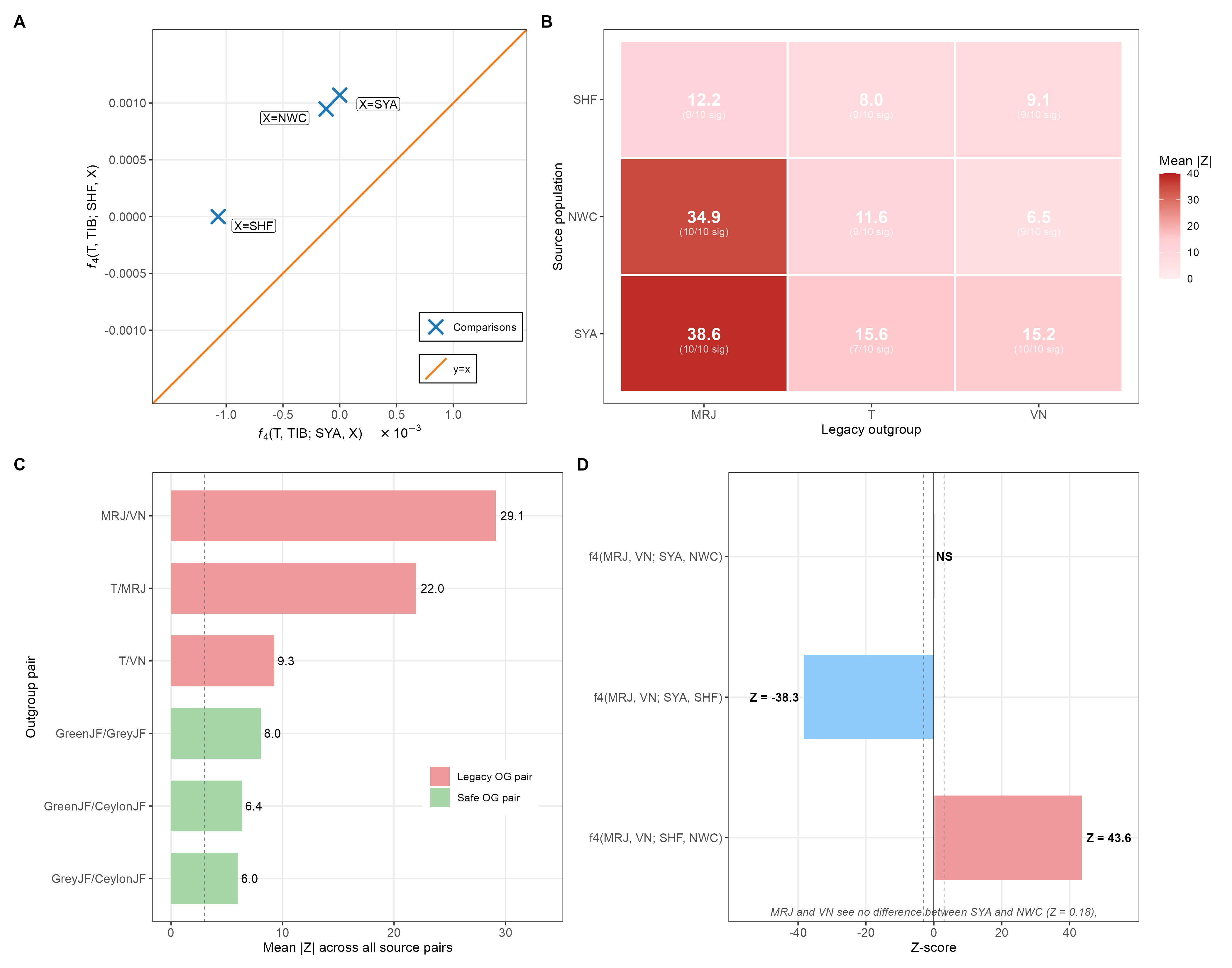


**Figure S3. Three-source model diagnostics: f4 symmetry and outgroup treeness.** (A) f4 symmetry test assessing model complexity: f4(T, TIB; SYA, X) vs f4(T, TIB; SHF, X) for X = {SHF, NWC, SYA}. If TIB were a simple two-way mixture, points would fall on the diagonal; all deviate significantly, rejecting any two-source model. (B) Treeness violation heatmap: mean |Z| of f4(GreenJF, Source; Legacy OG, TIB_sub) for each source–outgroup combination. All legacy outgroups show large violations (|Z| = 6.5–52.0). (C) Mean |Z| per outgroup pair across all source pairs. Legacy pairs (MRJ/VN, T/MRJ, T/VN) exceed |Z| > 3, whereas safe Gallus outgroup pairs remain below the threshold. (D) MRJ–VN asymmetry: f4(MRJ, VN; Src1, Src2) Z-scores for three key source-pair contrasts. MRJ and VN show no differential affinity between SYA and NWC (Z = 0.18, NS) but extreme asymmetry involving SHF (|Z| = 38–44), consistent with MRJ-mediated gene flow specific to the SHF lineage.

Statistical panels were generated in R or Python; composite layout and annotation were performed using the cowplot R package.

**Figure S4**


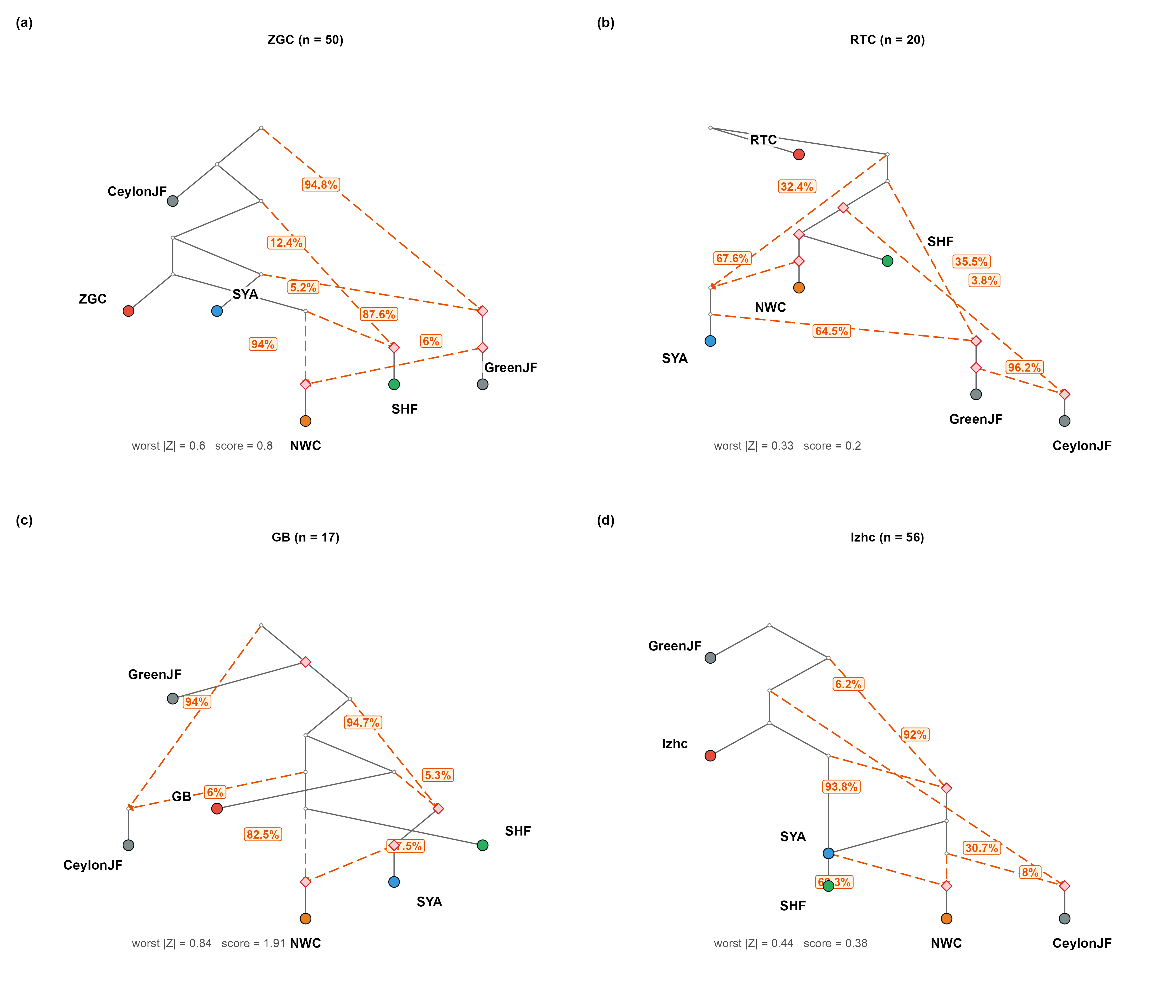


**Figure S4. Per-subpopulation qpGraph topologies.** Best-fit k = 3 admixture graph for each of the 10 TIB subpopulations, with worst |Z|-scores indicated. NWC is consistently modeled as an admixed node across all subpopulations, confirming this as a robust structural feature.

Statistical panels were generated in R or Python; composite layout and annotation were performed using the cowplot R package.

**Figure S5**


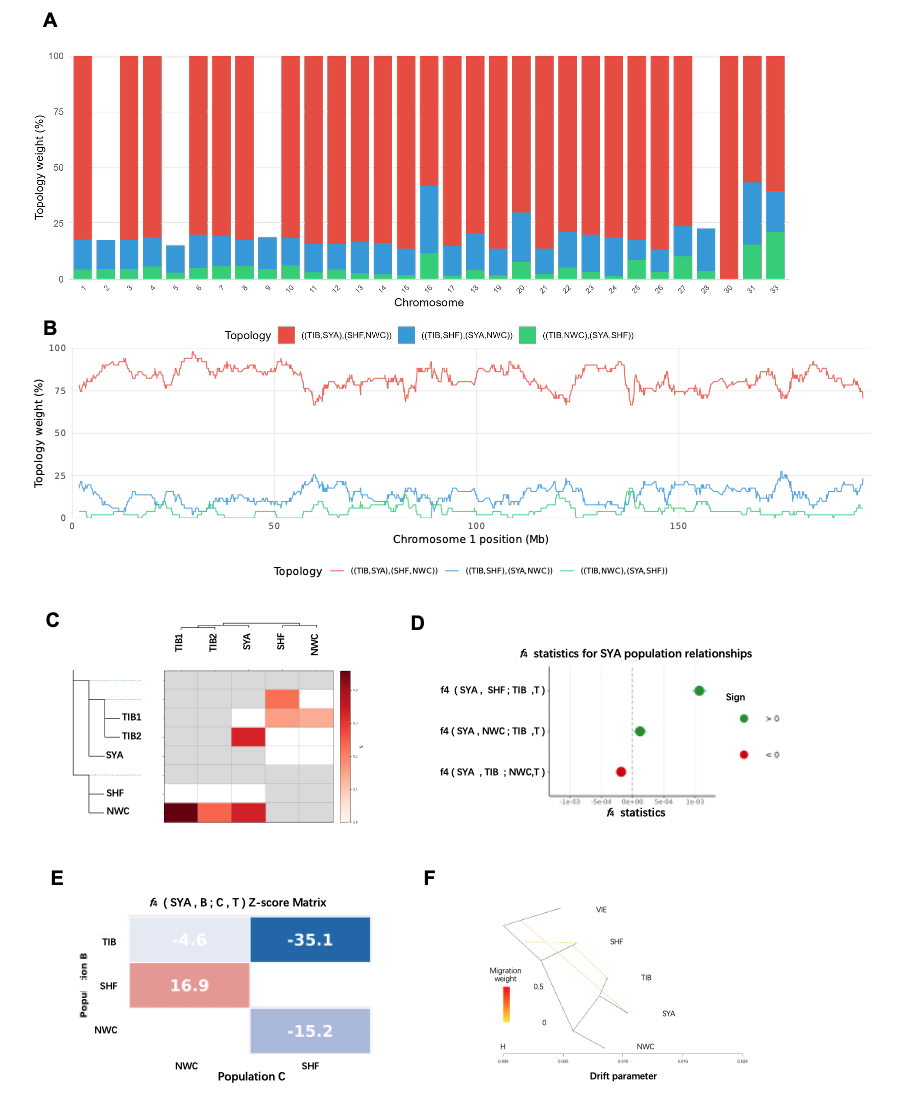


**Figure S5. Extended phylogenetic and f-statistic support.** (A) Per-chromosome Twisst topology weights. ((TIB, SYA), (SHF, NWC)) dominates across all chromosomes (range: 56.7–86.7%). Consensus-sequence approach: majority allele per population, PhyML BIONJ (HKY85), n = 11,243 valid windows genome-wide. (B) Sliding-window topology weights along chromosome 1. (C) f4 statistics centered on SYA: f4(SYA, B; C, T) for ingroup permutations. (D) Heatmap of Z-scores for f4(SYA, B; C, T). (E) F-branch clustering showing SYA grouped with TIB and away from T, supporting an artifact interpretation for the SYA→T TreeMix edge. (F) TreeMix population graph with VN outgroup (m = 3), confirming the SYA–TIB backbone under an alternative outgroup.

Statistical panels were generated in R or Python; composite layout and annotation were performed using the cowplot R package.

**Figure S6**


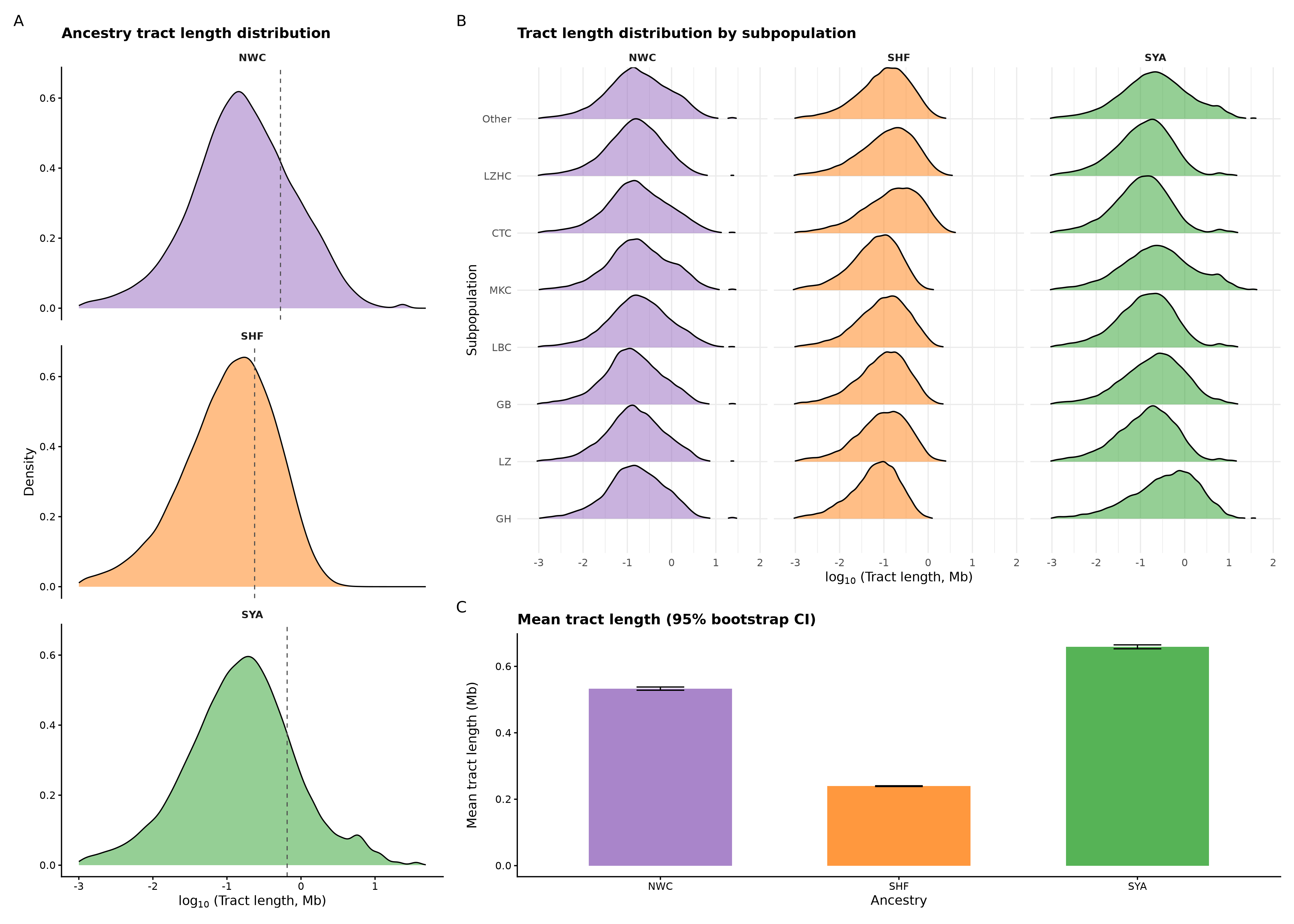


**Figure S6. Ancestry-specific tract-length distributions.** (A) Overall density of log10-transformed tract lengths. SHF shortest (mode ~0.1 Mb), NWC intermediate, SYA longest (mode ~1 Mb). (B) Distributions stratified by TIB subpopulation. (C) Mean tract length with 95% bootstrap CI (n = 1,000 resamples): SYA 0.65 Mb, NWC 0.53 Mb, SHF 0.24 Mb; all CIs non-overlapping.

Statistical panels were generated in R or Python; composite layout and annotation were performed using the cowplot R package.

**Figure S7**


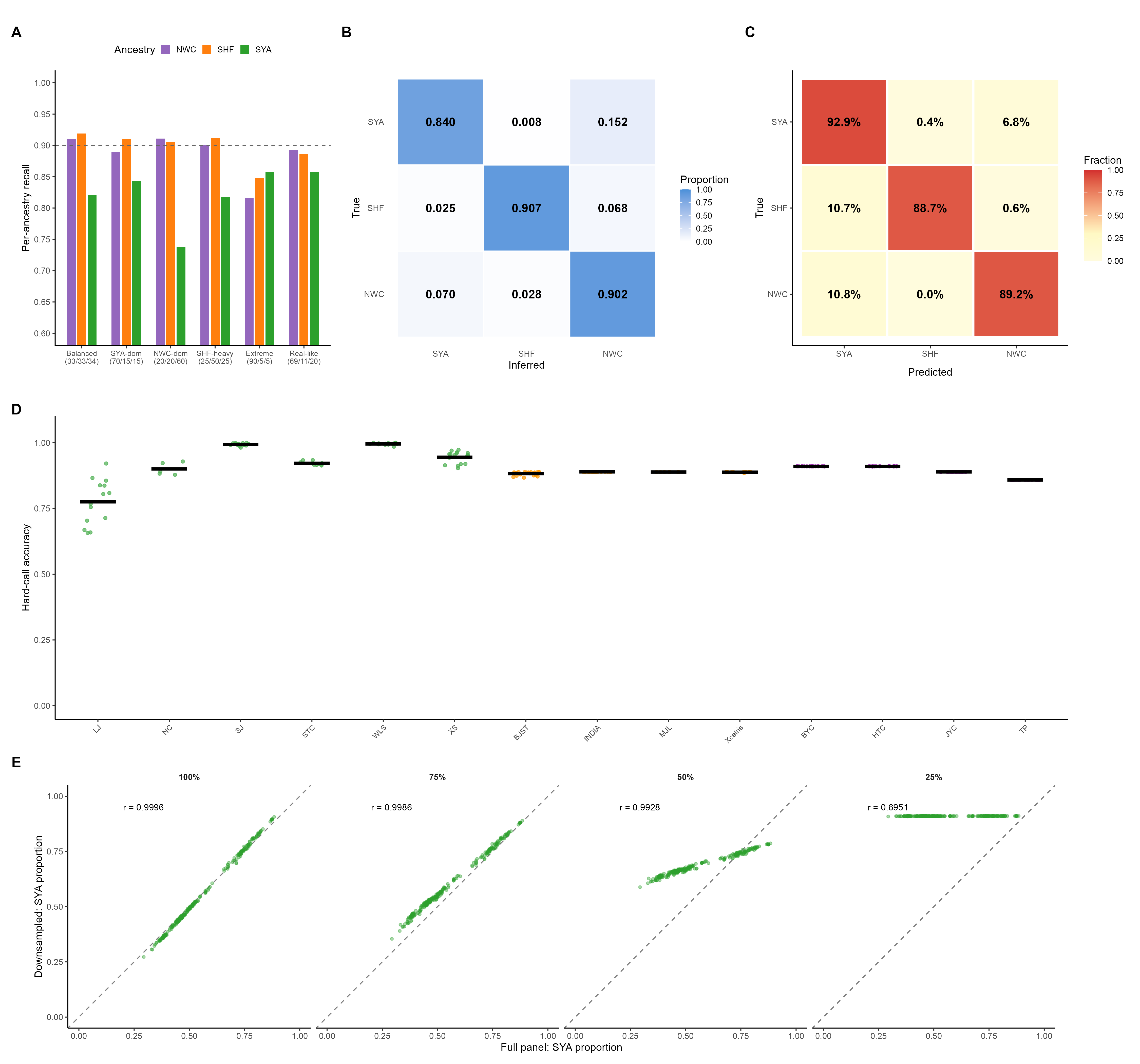


**Figure S7. LAI validation and robustness.** (A) Per-site recall for each ancestry across six pseudo-admixed simulation scenarios. (B) Confusion matrix from pseudo-admixed validation: dominant off-diagonal signal is SYA→NWC misassignment (15.2%). (C) Leave-one-out (LOO) confusion matrix (hard-call): SYA 92.9%, SHF 88.7%, NWC 89.2% correct. (D) Per-individual LOO hard-call accuracy across all breeds. (E) Downsampling sensitivity: per-individual SYA ancestry correlation between full and downsampled panels (Pearson r: 75% = 0.999, 50% = 0.993, 25% = 0.695).

Statistical panels were generated in R or Python; composite layout and annotation were performed using the cowplot R package.

**Figure S8**


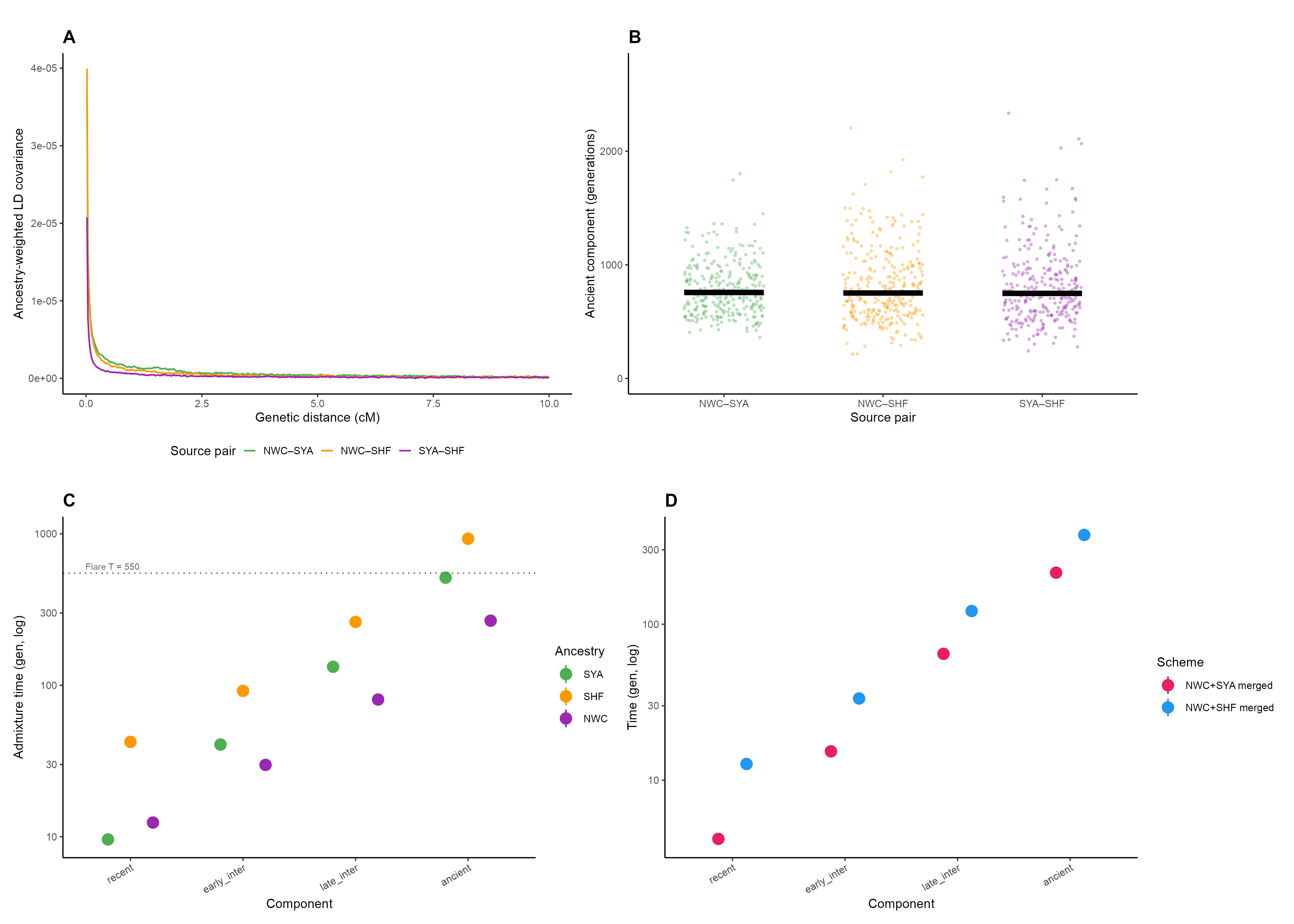


**Figure S8. Admixture dating: DATES LD decay and four-component tract-length estimates.** (A) Ancestry-weighted LD covariance curves for three DATES source-pair configurations (NWC–SYA, NWC–SHF, SYA–SHF; target = TIB), showing rapid decay within 2 cM superimposed on a slow-decaying ancient component. (B) Per-individual ancient-component estimates from two-exponential DATES fits across three source pairs (n = 284–292 per pair after QC; degenerate fits and >3×IQR outliers excluded). (C) Four-component (k = 4) truncated-exponential time estimates per ancestry (minT = 0.005 Mb). SHF ancient component (~928 gen) is the oldest; NWC apparent founding (~267 gen) is the youngest. (D) Binary reclassification validation. NWC+SYA merged ("non-SHF") and NWC+SHF merged ("non-SYA") each fitted with k = 4 model, confirming temporal structure is robust to ancestry label reassignment.

Statistical panels were generated in R or Python; composite layout and annotation were performed using the cowplot R package.

**Figure S9**


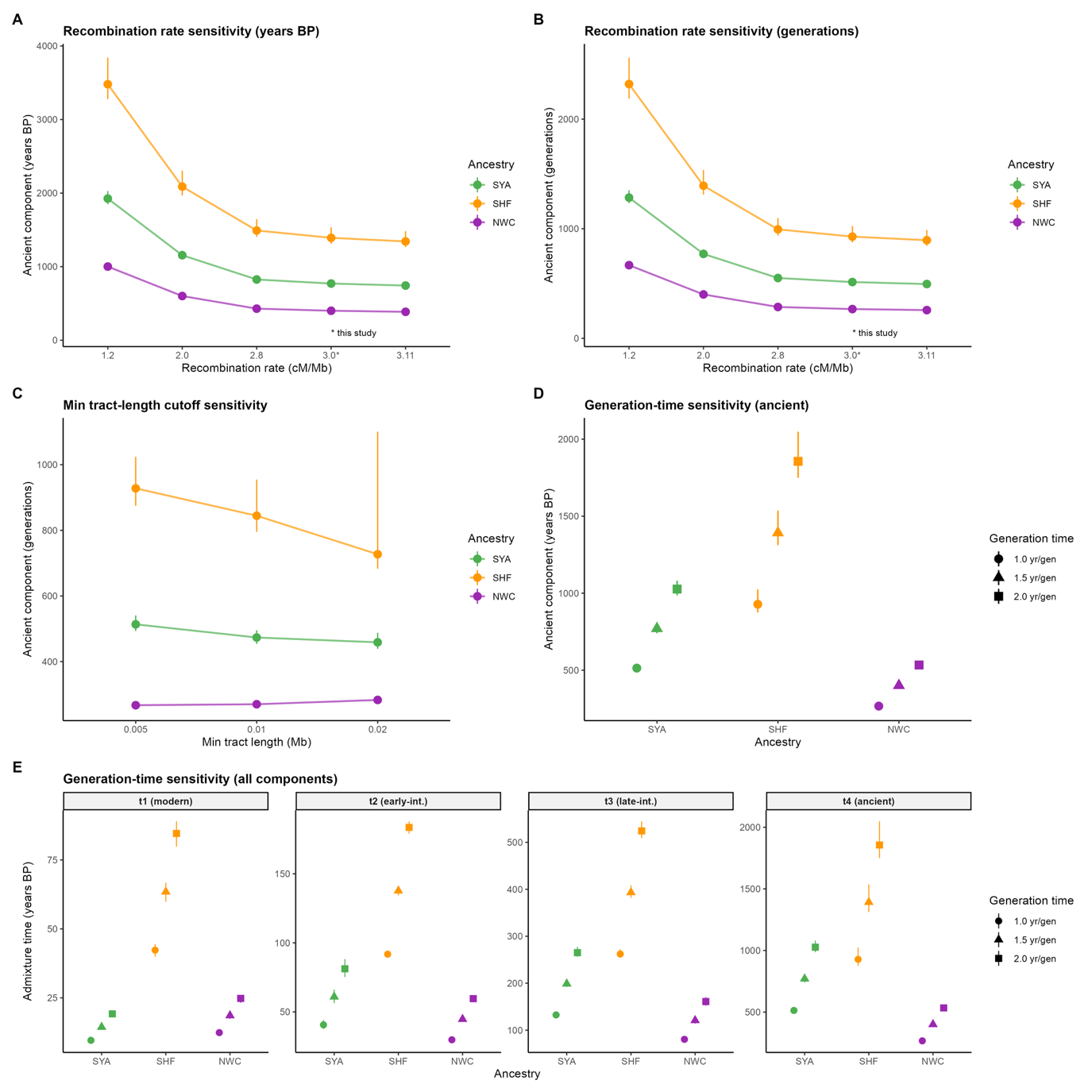


**Figure S9. Dating sensitivity analysis.** (A) Sensitivity of the ancient component to recombination rate (1.2–3.11 cM/Mb), shown as years BP (generation time = 1.5 yr/gen). The temporal ordering (SHF > SYA > NWC) is invariant across rates. (B) Same as (A), shown in generations. (C) Sensitivity of the ancient component to minimum tract-length cutoff (0.005–0.02 Mb). (D) Generation-time sensitivity for the ancient component under three assumptions (1.0, 1.5, 2.0 yr/gen). (E) Generation-time sensitivity for all four components, faceted by temporal tier.

Statistical panels were generated in R or Python; composite layout and annotation were performed using the cowplot R package.

**Figure S10**


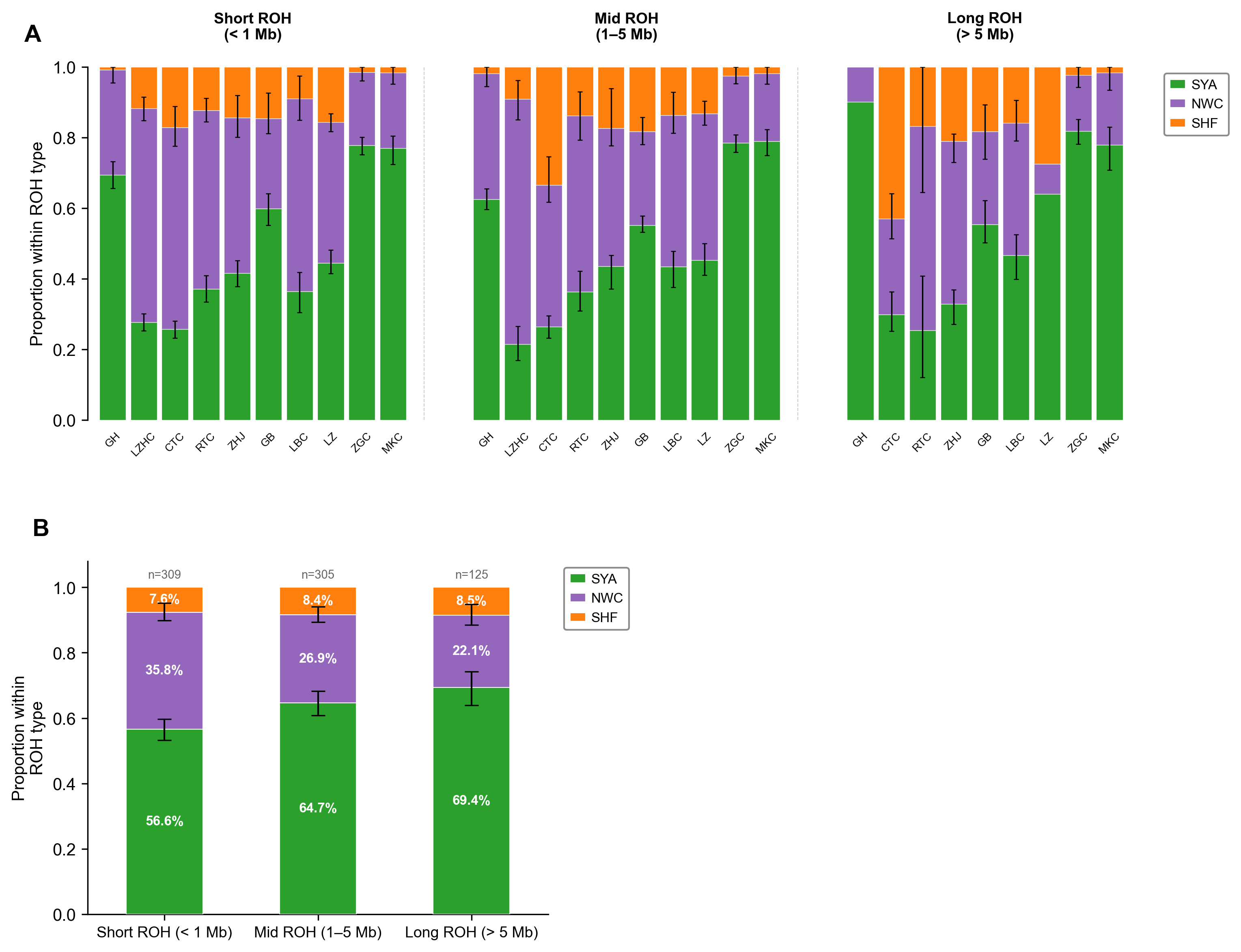


**Figure S10. Geographic clines of ancestry proportions and ROH stratification in Tibetan chickens.** Ancestry composition stratified by ROH length class. (A–C) Per-subpopulation ancestry proportions within short (A), mid (B), and long (C) ROH. (D) Genome-wide summary across all TIB individuals.

Statistical panels were generated in R or Python; composite layout and annotation were performed using the cowplot R package.

**Figure S11**


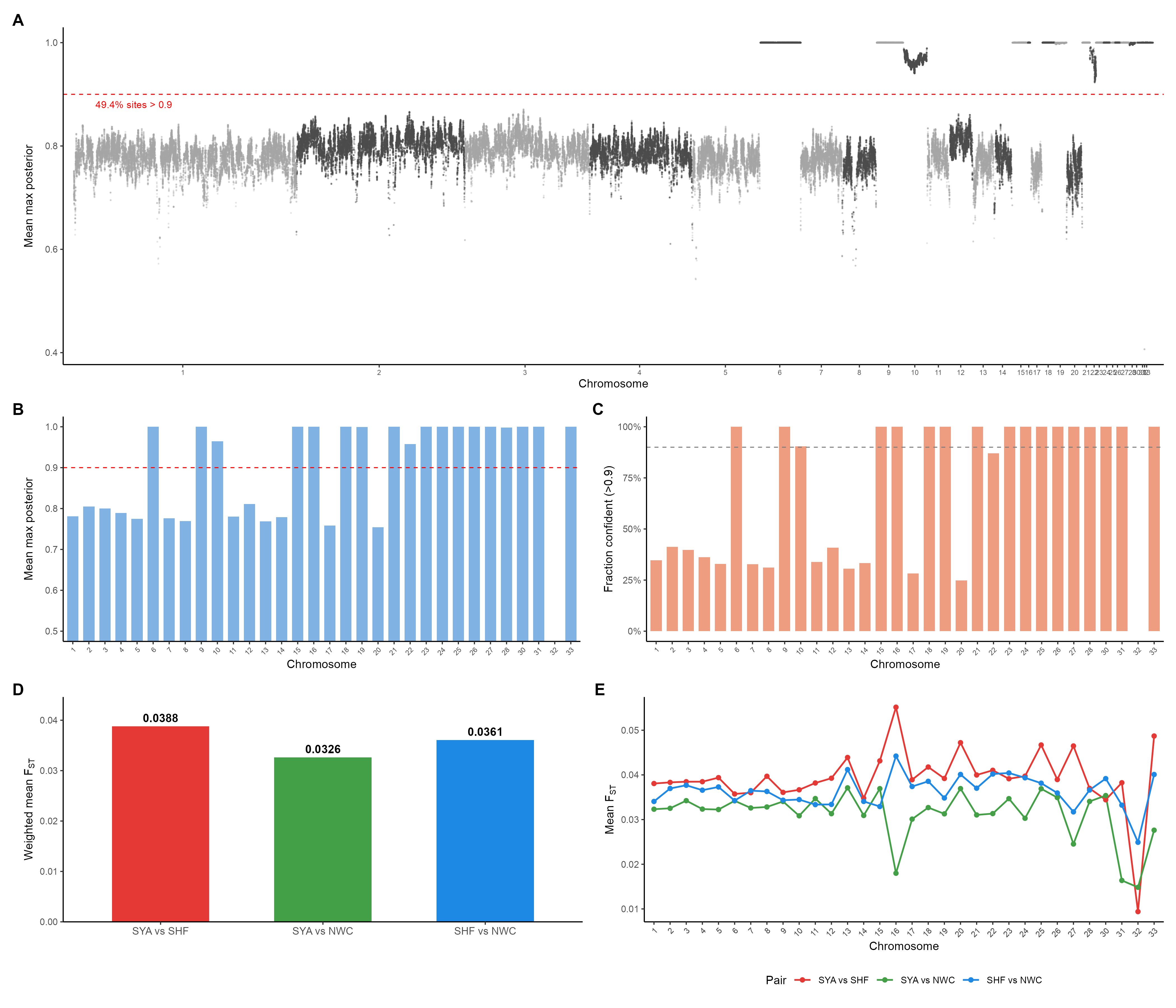


**Figure S11. LAI assignment confidence and reference-panel differentiation.** (A) Genome-wide Manhattan plot of mean max posterior probability across TIB individuals. Red dashed line: 0.9 threshold (49.4% above). (B) Per-chromosome mean max posterior. (C) Fraction of sites per chromosome exceeding the 0.9 confidence threshold: macrochromosomes 25–35%; microchromosomes > 90%. (D) Genome-wide weighted-mean F_ST between reference panels: SYA–NWC = 0.0326, SHF–NWC = 0.0361, SYA–SHF = 0.0388. (E) Per-chromosome mean F_ST. SYA–NWC shows pronounced dips on several microchromosomes (e.g., chr16, chr31–33), coinciding with regions of lower assignment confidence.

Statistical panels were generated in R or Python; composite layout and annotation were performed using the cowplot R package.

**Figure S12**


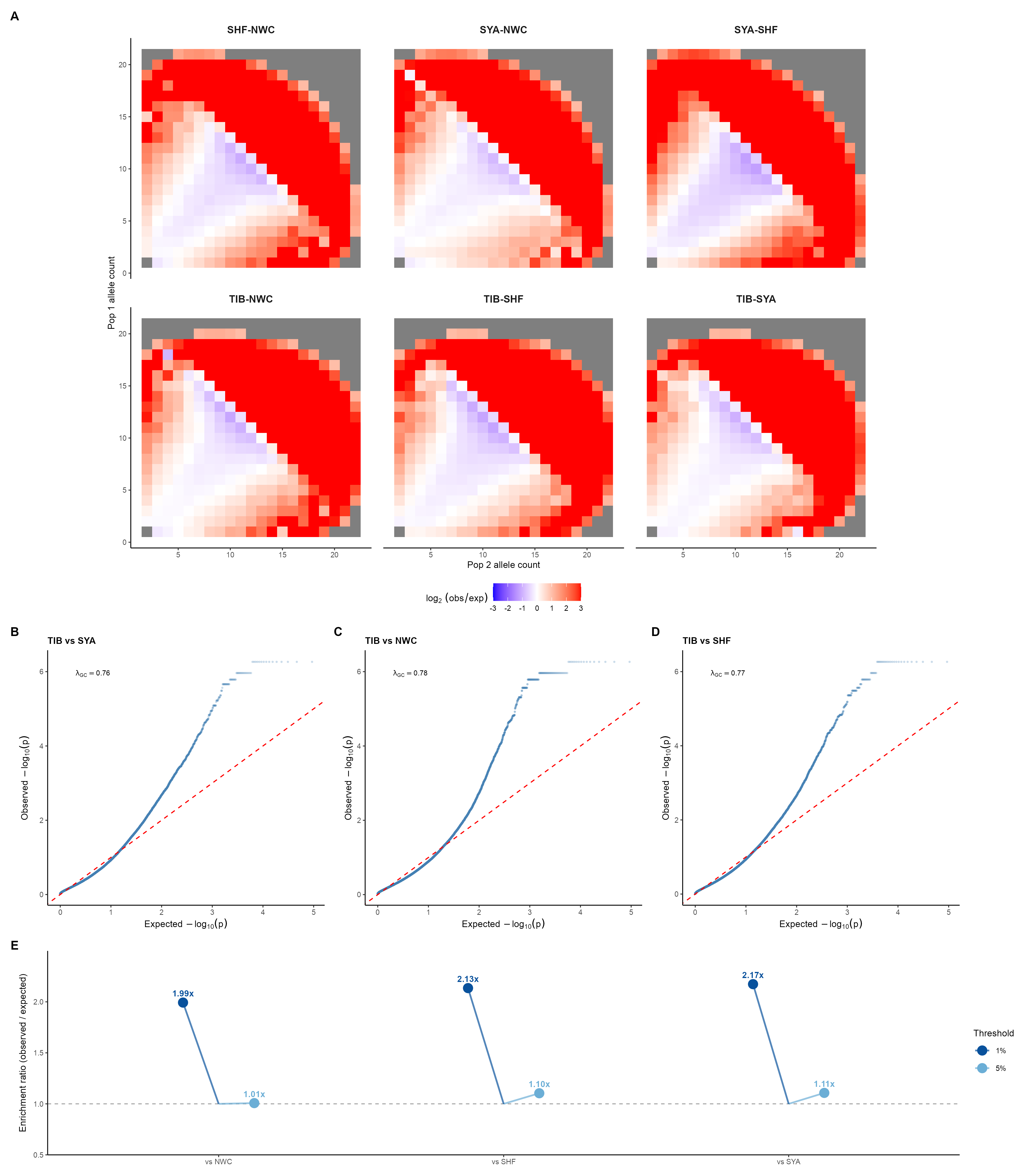


Figure S12. Neutral demographic null validation. (A) Observed versus expected 2D folded SFS residuals under the best-fit Pulse model for six population pairs. Color: log2 fold-change (red: observed excess; blue: deficit). Good fit at intermediate frequencies; excess rare variants at SFS edges confirms a conservative null. (B–D) Quantile–quantile plots of calibrated F_ST p-values for TIB vs SYA (B), TIB vs NWC (C), and TIB vs SHF (D). λ_GC = 0.76–0.78, indicating conservative null. (E) Tail enrichment: observed-to-expected fraction of windows exceeding neutral 5% and 1% thresholds. At 1%: 1.99x (NWC), 2.13x (SHF), 2.17x (SYA).

Statistical panels were generated in R or Python; composite layout and annotation were performed using the cowplot R package.

**Figure S13**


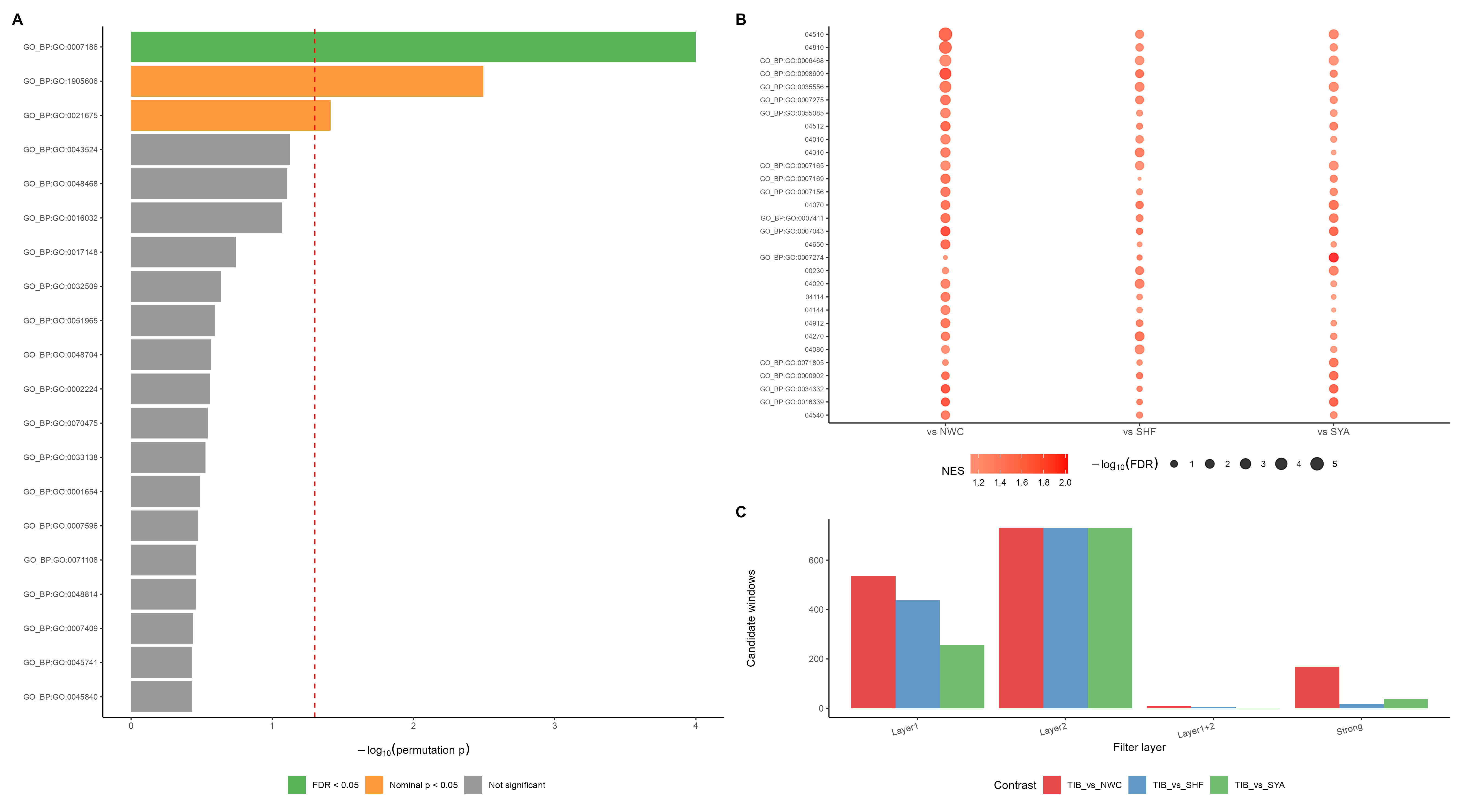


**Figure S13. Enrichment analysis and candidate filtering pipeline.** (A) Matched-region permutation enrichment test: top 20 pathways by permutation p-value (10,000 iterations; controlling for gene density and GC content). Only GPCR signaling (GO:0007186) survived FDR correction (FDR = 0.030; fold-change = 2.19x). (B) Gene-set enrichment analysis (GSEA) dot plot across ancestry contrasts: top 30 pathways by NES, with FDR indicated by point size. (C) Three-layer candidate filtering summary: Layer 1 (FDR q < 0.05): NWC = 536, SHF = 437, SYA = 255; Strong candidates (all layers): NWC = 169, SHF = 17, SYA = 37 (total 223).

Statistical panels were generated in R or Python; composite layout and annotation were performed using the cowplot R package.

**Figure S14**


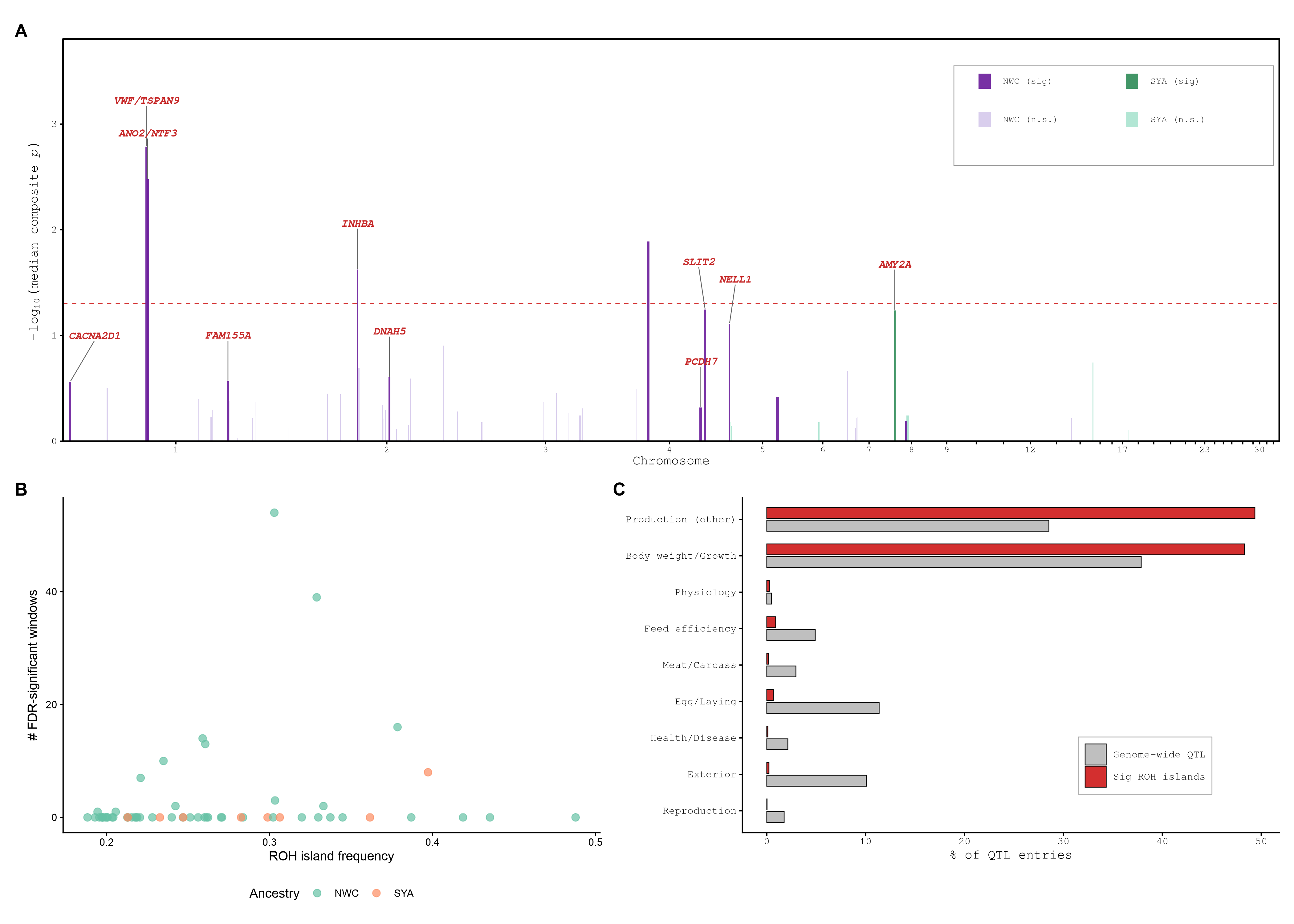


**Figure S14. ROH islands: selection signals and QTL annotation.** (A) Genome-wide ROH Manhattan plot with ancestry-labeled islands. Purple: NWC-ancestry; green: SYA-ancestry. Filled colors: islands harboring FDR-significant selection windows (13/58); lighter shades: non-significant. (B) FDR-significant windows vs. ROH frequency scatter. NWC: 8.36x F_ST enrichment (OR = 8.72, p = 3.0 x 10^-83). (C) QTL category distribution in significant ROH islands versus genome-wide (Animal QTLdb Release 58). Body-weight/growth QTLs enriched (OR = 13.1, FDR = 7.6 × 10^−22).

Statistical panels were generated in R or Python; composite layout and annotation were performed using the cowplot R package.
